# Functional polyamine metabolic enzymes and pathways encoded by the virosphere

**DOI:** 10.1073/pnas.2214165120

**Published:** 2023-02-21

**Authors:** Bin Li, Jue Liang, Hamid R. Baniasadi, Margaret A. Phillips, Anthony J. Michael

**Affiliations:** ^a^Department of Biochemistry, University of Texas Southwestern Medical Center, Dallas, TX 75214

**Keywords:** polyamine, spermidine, putrescine, virus, bacteriophage

## Abstract

Viruses produce more viruses by manipulating the metabolic and replication systems of their host cells. Many have acquired metabolic genes from ancestral hosts and use the encoded enzymes to subvert host metabolism. The polyamine spermidine is required for bacteriophage and eukaryotic virus replication, and herein, we have identified and functionally characterized diverse phage- and virus-encoded polyamine metabolic enzymes and pathways. These include pyridoxal 5′-phosphate (PLP)-dependent ornithine decarboxylase (ODC), pyruvoyl-dependent ODC and arginine decarboxylase (ADC), arginase, *S*-adenosylmethionine decarboxylase (AdoMetDC/*speD*), spermidine synthase, homospermidine synthase, spermidine *N*-acetyltransferase, and *N*-acetylspermidine amidohydrolase. We identified homologs of the spermidine-modified translation factor eIF5a encoded by giant viruses of the *Imitervirales*. Although AdoMetDC/*speD* is prevalent among marine phages, some homologs have lost AdoMetDC activity and have evolved into pyruvoyl-dependent ADC or ODC. The pelagiphages that encode the pyruvoyl-dependent ADCs infect the abundant ocean bacterium *Candidatus Pelagibacter ubique*, which we have found encodes a PLP-dependent ODC homolog that has evolved into an ADC, indicating that infected cells would contain both PLP- and pyruvoyl-dependent ADCs. Complete or partial spermidine or homospermidine biosynthetic pathways are found encoded in the giant viruses of the *Algavirales* and *Imitervirales*, and in addition, some viruses of the *Imitervirales* can release spermidine from the inactive *N*-acetylspermidine. In contrast, diverse phages encode spermidine *N*-acetyltransferase that can sequester spermidine into its inactive *N*-acetyl form. Together, the virome-encoded enzymes and pathways for biosynthesis and release or biochemical sequestration of spermidine or its structural analog homospermidine consolidate and expand evidence supporting an important and global role of spermidine in virus biology.

## Significance

Viruses require the small polyamine spermidine to replicate normally, in part due to its important role in ribosomal translation. We found genes in many viruses of bacteria (bacteriophages) and eukaryotic viruses encoding enzymes that produce spermidine or the structurally similar homospermidine or that release spermidine from an inactive form or render it into an inactive form. Some viruses encode single polyamine metabolic enzymes, while others encode entire or partial metabolic pathways. By biochemically analyzing bacteriophage enzymes that lacked expected activity, we discovered that the enzymes have evolved to recognize different polyamine-related substrates. Giant viruses that are known to encode many translation-related proteins encode the most complete polyamine metabolic pathways, consistent with an important role of polyamines in virus replication.

**Fig. 1.**
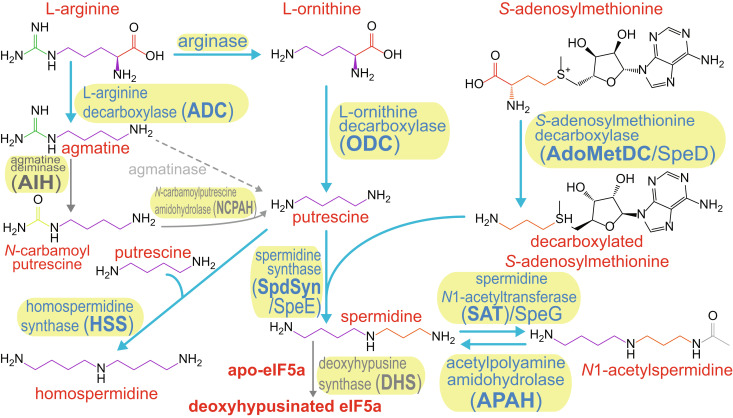
Polyamine metabolic pathways. Pathways biochemically characterized herein are indicated by blue arrows and blue enzyme names.

The polyamine spermidine (Fig. 1) is a metabolically primordial polycation found throughout bacteria, archaea, and eukaryotes (1). It is a fundamental molecule of life that was likely present in the last universal common ancestor (2). In *Escherichia coli*, 90% of spermidine is noncovalently bound to RNA (3) and is required for efficient translational elongation by the ribosome (4). Spermidine increases global messenger RNA (mRNA) translation in *E. coli* by facilitating the queuosine modification of specific tRNA anticodon wobble bases (4). Consistent with these findings, in strains of *E. coli* deleted for genes that modify the anticodon wobble position in transfer RNAs (tRNAs), spermidine becomes absolutely essential for growth (5), which may be due to spermidine-mediated stabilization of the tRNA interaction with the translating ribosome. Spermidine is not only important for growth of bacteria; over 40 y ago, it was shown that T4 and T7 bacteriophages replicated more slowly in a spermidine-deficient mutant of *E. coli* (6). Replication of JG004 and N4-like phages in *Pseudomonas aeruginosa* PAO1 is absolutely dependent on spermidine (7, 8).

In eukaryotic cells, spermidine is universally required for growth and cell proliferation. An aminobutyl moiety of spermidine (Fig. 1) is transferred by deoxyhypusine synthase (DHS) to a single lysine of the translation factor eIF5A to eventually form the essential hypusine posttranslational modification (9). Hypusinated eIF5a is needed for translation of mRNAs encoding proline-rich motifs and for translation termination (10). Replication of eukaryotic RNA viruses is highly dependent on host spermidine (11), and spermidine-derived hypusination of host eIF5a is required for Ebola virus replication and is considered a potential target to inhibit viral replication (12).

Viruses reprogram the metabolism of host cells to make more virions by redirecting expression and activity of host-encoded enzymes and by expressing virus-encoded enzymes. Using homology-based approaches, nucleocytoplasmic large DNA viruses have been found to encode homologs of enzymes involved in nitrogen metabolism, glycolysis, and the tricarboxylic acid cycle (13). Bacteriophages have been found to encode homologs of enzymes involved in inorganic sulfur metabolism (14) and nucleotide metabolism (15). The eukaryotic chlorovirus *Paramecium bursaria* chlorella virus 1 (PBCV-1) encodes an entire functional biosynthetic pathway for production of homospermidine (Fig. 1), a structural analog of spermidine, consisting of L-arginine decarboxylase (ADC), agmatine deiminase/iminohydrolase (AIH), *N*-carbamoylputrescine amidohydrolase (NCPAH), and homospermidine synthase (HSS) (16–18). In addition, PBCV-1 encodes a polyamine *N*-acetyltransferase (19). A biochemically functional HSS enzyme is encoded by *Ralstonia* phage ϕRSL1 (20). Considering the importance of polyamines to phage and virus replication, we sought to systematically identify and functionally characterize polyamine metabolic enzymes and pathways encoded in phage and virus genomes. Some of the taxonomic affiliations of giant viruses included in our study are based on a recently published hierarchical taxonomy for the Nucleocytoviricota (21).

## Results and Discussion

### Virus-Encoded Putrescine Biosynthesis.

Ornithine decarboxylase (ODC) is the first and a rate-limiting step in eukaryotic polyamine biosynthesis converting L-ornithine directly to putrescine (Fig. 1). The form of ODC encoded by eukaryotic species is a pyridoxal 5′-phosphate (PLP)–dependent protein belonging to the alanine racemase (AR) fold, and its inhibition in host cells by the irreversible inhibitor α-difluoromethylornithine is known to inhibit replication of DNA and RNA viruses (22). Attributing enzymatic function to homologs of ODC is complicated by the fact that ODC belongs to a family of homologous enzymes acting on diverse substrates. These include decarboxylases acting on *meso*-diaminopimelate (DAPDC), L-arginine (ADC), L-carboxyspermidine, L-lysine and L-ornithine (L/ODC), and more specialized substrates such as *N*-citrylornithine (23). Using Protein Basic Local Alignment Search Tool (BLASTP) and Position Specific Iterative BLAST (PSI-BLAST), we identified potential ODCs encoded in eukaryotic virus genomes from the *Imitervirales* and *Algavirales* orders and *Herpesviridae* and *Poxviridae* families (*SI Appendix*, Table S1). Although we did not detect likely ODC homologs in phage genomes, we did detect DAPDC (*SI Appendix*, Table S1), which is the ultimate biosynthetic step for L-lysine and penultimate biosynthetic step for diamine cadaverine in bacteria. To determine the likelihood of the homologs being bona fide ODCs, we constructed a phylogenetic tree (*SI Appendix*, *Supplementary Experimental Section*) of the virus proteins together with diverse AR-fold decarboxylases of known function (*SI Appendix*, Fig. S1). The virus ODCs were limited to a robustly supported clade containing biochemically validated bacterial and eukaryotic ODCs, bifunctional L/ODCs, and ODC proteins that have recently evolved into ADCs. The phage DAPDC homologs were also limited to a strongly supported clade containing validated DAPDCs except for one *Podoviridae* phage protein that was found in a clade of specialized metabolism enzymes.

ODC homologs from five viruses were selected for expression in *E. coli*, protein purification, and kinetic analysis (*SI Appendix*, *Supplementary Experimental Section*). Genes from *Cafeteria roenbergensis* virus and bovine gammaherpesvirus 6 (*SI Appendix*, Table S1) produced proteins that were completely insoluble when expressed in *E. coli*, whereas those from Yellowstone Lake phycodnavirus 2 (*Algavirales*), Klosneuvirus KNV1, and Tupanvirus soda lake (*Imitervirales*) produced soluble protein that was then purified and assayed for decarboxylase activity with L-ornithine, L-lysine, and L-arginine (Table 1). No activity on L-arginine was detected with any protein, and L-ornithine was greatly favored over L-lysine by all three proteins, consistent with them being bona fide ODCs. The virus ODCs were aligned with diverse, functionally validated eukaryotic ODCs, and ODC homologs from the major eukaryotic phyla to determine whether different virus ODCs were acquired independently from different hosts (*SI Appendix*, Fig. S2). Virus ODC homologs are found in at least five discrete clades, and notably, the bovine gammaherpesvirus ODC is found in a clade containing vertebrate ODCs. A group of *Imitervirales* virus ODCs encoded in Yasminevirus sp. GU-2018, Terrestrivirus 2_66, Tupanvirus soda lake, Tupanvirus deep ocean, Fadolivirus 1, Klosneuvirus KNV1, and phycodnaviruses Dishui lakes 2, 3, and 4 are found together in a robustly supported virus-only clade. A separate clade contains chlorovirus ODC-like ADC (*Algavirales*) and Yellowstone Lake phycodnavirus ODCs and is closely related to plant and green algal ODCs. The *Cafeteria roenbergensis* virus ODC homolog is found separately from all other viral ODCs, as is the Organic Lake phycodnavirus ODC. This phylogenetic evidence suggests that ODC has been acquired independently by viruses at least five times and from different hosts.

**Table 1.** Kinetic parameters for virus ornithine decarboxylase homologs

Virus protein	Substrate	*K_M_* (mM)	*k*_cat_ (s^1^)	*k*_cat_/*K_M_* (s^1^ M^1^)
Klosneuvirus KNV1	L-ornithine	0.43 ± 0.02	0.48 ± 0.040	1,120 ± 31
Klosneuvirus KNV1	L-lysine	43 ± 14	0.86 ± 0.22	20 ± 1.4
Klosneuvirus KNV1	L-arginine	n.a.	n.a.	n.a.
Tupanvirus soda lake	L-ornithine	1.1 ± 0.16	0.47 ± 0.070	450 ± 32
Tupanvirus soda lake	L-lysine	13 ± 3.1	0.090 ± 0.020	6.9 ± 0.96
Tupanvirus soda lake	L-arginine	n.a.	n.a.	n.a.
Yellowstone Lake phycodnavirus 2	L-ornithine	0.54 ± 0.20	0.14 ± 0.0030	260 ± 4.3
Yellowstone Lake phycodnavirus 2	L-lysine	13 ± 0.66	0.28 ± 0.012	22 ± 0.84
Yellowstone Lake phycodnavirus 2	L-arginine	n.a.	n.a.	n.a.

Assayed at 26°C (±SD of three replicate assays). Klosneuvirus KNV1 (ARF12269; 420 aa), Tupanvirus soda lake (QKU35264; 440 aa), and Yellowstone Lake phycodnavirus 2 (YP_009174582; 376 aa). n.a. (no detectable activity).

We detected neither any phage or virus homologs of the typical AR-fold ADC represented by the *E. coli* or *Arabidopsis thaliana* ADCs nor any of the bacterial aspartate aminotransferase-fold ADCs (24). It was shown previously that the chlorovirus PBCV-1 encodes an ODC homolog that has recently evolved into an ADC (17). The chlorovirus ADC produces agmatine from arginine (Fig. 1), and agmatine is then converted to putrescine by AIH and NCPAH encoded by PBCV-1 (18). Although the ODC-like ADC and NCPAH are encoded in 40 chlorovirus genomes (*SI Appendix*, Table S2), only 28 of those genomes encode a complete AIH, indicating that putrescine biosynthesis is dispensable for replication of some chlorovirus strains. However, all chlorovirus genomes encode an intact HSS, suggesting that some chlorovirus strains rely on host-supplied putrescine to synthesize homospermidine. We did not detect any AIH or NCPAH homologs in viruses outside the chloroviruses, consistent with the other virus ODC homologs acting on L-ornithine rather than L-arginine.

The availability of L-ornithine can be limiting for putrescine biosynthesis (25). Arginase, a manganese-dependent enzyme that converts L-arginine to L-ornithine and urea, belongs to an enzyme family that also includes agmatinase, an enzyme that converts agmatine to putrescine (26). The L-ornithine product of arginase is the substrate for ODC (Fig. 1). Virus proteomes were screened for arginase homologs using BLASTP. Homologs representing potential orthologs were detected in 11 genomes from the *Imitervirales*, one from the *Algavirales* but none in phage (*SI Appendix*, Table S3). Four of the *Imitervirales* genomes encoding a homolog of arginase also encoded ODC: Klosneuvirus KNV1, Terrestrivirus sp. [partial open reading frame (ORF)], Yasminevirus sp. GU-2018, and Fadolivirus 1, which encodes two arginase homologs. No obvious homologs of agmatinase were detected in virus genomes, and only one was detected in a phage genome, an uncultured Caudovirales phage (CAB4123030; 303 aa).

To determine whether virus arginase homologs exhibit arginase enzymatic activity, we chose to test the gene from Klosneuvirus KNV1. After expression and purification in *E. coli*, the KNV1 arginase homolog was assayed with L-arginine or agmatine as a substrate using an assay based on detection of released urea. No activity was detected with agmatine, but activity with L-arginine showed a *K*_m_ of 1.3 ± 0.16 mM and *k*_cat_ of 2.8 ± 0.05 s^−1^, with a *k*_cat_/*K*_m_ of 2.1 × 10^4^ M^−1^ s^−1^. In an orthogonal approach to confirm arginase activity, the L-ornithine product of the Klosneuvirus enzyme was detected by Liquid Chromatography Tandem Mass Spectrometry (LC–MS/MS) (*SI Appendix*, Fig. S3). A low level of L-ornithine is present in the reaction with the boiled enzyme, which is derived from a low level of L-ornithine in the L-arginine stock, but the active enzyme produced an LC–MS/MS extracted ion chromatogram peak three orders of magnitude greater than the boiled enzyme. Together, these data show that the Klosneuvirus arginase homolog is a highly specific and efficient arginase.

### Virus-Encoded Spermidine Biosynthesis.

A second rate-limiting step in spermidine biosynthesis is performed by *S*-adenosylmethionine decarboxylase (AdoMetDC/SpeD), a pyruvoyl cofactor-dependent enzyme that undergoes autocatalytic self-processing at a serine residue to form the covalently attached pyruvoyl cofactor (27). There are three structurally related forms of AdoMetDC: class 1b, the smallest and most common in prokaryotes, class 1a typified by the *E. coli* AdoMetDC, which is derived from 1b and is larger, and the eukaryotic-type class 2 (28). We did not detect by BLASTP and PSI-BLAST any class 1a or class 2 AdoMetDCs encoded by viruses or phages but identified diverse class 1b homologs. To determine whether phage and virus AdoMetDC homologs were functional, the corresponding open reading frames (ORFs) were expressed in a spermidine-deficient AdoMetDC (*speD*) deletion strain of *E. coli* (BL21Δ*speD*). The *E. coli* AdoMetDC/*speD* ORF is immediately downstream of the spermidine synthase (SpdSyn/*speE*) ORF, which is required for transfer of the aminopropyl group from decarboxylated *S*-adenosylmethionine to putrescine for spermidine biosynthesis. To ensure that full SpdSyn/SpeE activity was maintained in the BL21Δ*speD* deletion strain, the *E. coli speE* ORF was coexpressed with the phage and virus *speD* ORFs. Restoration of spermidine accumulation confirmed bona fide AdoMetDC function for homologs from *Synechococcus* phage S-PM2, *Thermus* phage phiYs40, *Sinorhizobium* phage phiN3, *Croceibacter* phage P2559s, and eukaryotic viruses of the *Algavirales*: *Heterosigma akashiwo* virus 01 and Yellowstone Lake phycodnavirus 2 (*SI Appendix*, Fig. S4). The AdoMetDC-encoding phages infect hosts from diverse bacterial phyla: Cyanobacteria, Deinococcus-Thermus, α-Proteobacteria, and Bacteroidetes. It is notable that the eukaryotic viruses encode prokaryotic AdoMetDCs.

We were able to determine that the *E. coli* BL21Δ*speD* strain retains a functional *speE* gene; however, an AdoMetDC homolog (AHX71538) from pelagiphage HTVC201P did not restore spermidine accumulation to BL21Δ*speD* when the positive control *speD* from *Bacillus subtilis* did (*SI Appendix*, Fig. S5). The host of HTVC201P is found in α-Proteobacteria SAR11 bacteria, in particular *Candidatus Pelagibacter ubique*, the most abundant ocean bacterial species (29). We suspected that the AdoMetDC homolog may have shifted substrate preference, and therefore purified the recombinant HTVC201P AdoMetDC protein, and assayed it with L-ornithine, L-lysine, and L-arginine (Table 2). No decarboxylation activity was found with L-ornithine or L-lysine, but the protein decarboxylated L-arginine with a *K*_m_ of 0.87 ± 0.11 mM, *k*_cat_ of 0.25 ± 0.010 s^−1^, and *k*_cat_/*K*_m_ of 290 ± 32 M^−1^ s^−1^ at 27 °C. The only known example of an AdoMetDC homolog that has evolved into an ADC is from the Crenarchaeote *Sulfolobus solfataricus*, and it exhibits a relatively higher *k*_cat_/*K*_m_ with arginine of 1.3 × 10^4^ M^−1^ s^−1^, although this activity was obtained at 70 °C (30). However, the nonhomologous pyruvoyl-dependent ADC of *Methanocaldococcus jannaschii* (a pyruvoyl-dependent enzyme from a different structural fold to AdoMetDC) had a *k*_cat_/*K*_m_ of 380 M^−1^ s^−1^ at 83 °C (31), which is more similar to the efficiency of the HTVC201P AdoMetDC. Enzymes exhibiting the same activity but arising from different protein folds have been termed nonhomologous isofunctional enzymes (32). A protein (BAR31446) with close homology to the HTVC201P AdoMetDC/ADC was found to be encoded by an uncultivated marine phage. It did not restore spermidine accumulation to BL21Δ*speD* (*SI Appendix*, Fig. S6*A*), and the purified recombinant protein was completely insoluble. However, when expressed at a lower level in *E. coli*, we detected a threefold higher peak for the product of arginine decarboxylation, agmatine, by Liquid Chromatography-Mass Spectrometry (LC–MS) analysis (*SI Appendix*, Fig. S6*B*), consistent with the AdoMetDC homolog BAR31446 being a pyruvoyl-dependent ADC.

**Table 2.** Kinetic parameters for phage *S*-adenosylmethionine decarboxylase and virus ornithine decarboxylase homologs that have evolved into pyruvoyl- and PLP-dependent arginine decarboxylases

Virus protein	Substrate	*K_M_*(mM)	*k*_cat_ (s^1^)	*k*_cat_/*K_M_*(s^1^ M^1^)
Pelagiphage HTVC201P “AdoMetDC”	L-ornithine	n.a.	n.a.	n.a.
Pelagiphage HTVC201P “AdoMetDC”	L-lysine	n.a.	n.a.	n.a.
Pelagiphage HTVC201P “AdoMetDC”	L-arginine	0.87 ± 0.11	0.25 ± 0.010	290 ± 32
*Ca. Pelagibacter ubique* “ODC”	L-ornithine	n.a.	n.a.	n.a.
*Ca. Pelagibacter ubique* “ODC”	L-lysine	n.a.	n.a.	n.a.
*Ca. Pelagibacter ubique* “ODC”	L-arginine	2.7 ± 0.12	0.32 ± 0.0010	120 ± 5.5
*Ca. Fonsibacter ubiquis* “ODC”	L-ornithine	n.a.	n.a.	n.a.
*Ca. Fonsibacter ubiquis* “ODC”	L-lysine	n.a.	n.a.	n.a.
*Ca. Fonsibacter ubiquis* “ODC”	L-arginine	1.3 ± 0.12	2.2 ± 0.13	1,700 ± 91

Assayed at 27 °C (±SD of three replicate assays). Pelagiphage HTVC201P “AdoMetDC” (AXH71538; 122 aa), *Ca. Pelagibacter ubique* “ODC” (WP_075506504; 394 aa), and *Ca. Fonsibacter ubiquis* “ODC” (WP_099339815; 396 aa). n.a. (no detectable activity).

A natural host of pelagiphage HTVC201P, which as we have demonstrated encodes an AdoMetDC-derived pyruvoyl-dependent ADC, is *Ca. Pelagibacter* HTCC7211 belonging to the *Pelagibacterales* order of the α-Proteobacteria (33). In most α-Proteobacteria, L-ornithine is converted to putrescine by an AR-fold PLP-dependent ODC, and ADC along with the enzymes responsible for converting agmatine to putrescine are considerably less common. This suggests that the agmatine produced by the pelagiphage pyruvoyl-dependent ADC would not have a host-encoded biosynthetic route from agmatine to putrescine. We decided to analyze the AR-fold ODC homologs from *Ca. P. ubique* SCGC AAA797-I19 and the freshwater *Pelagibacterales* lineage *Candidatus Fonsibacter ubiquis* LSUCC0530. After purification of the recombinant *Pelagibacter* and *Fonsibacter* enzymes from *E. coli*, both ODC homologs (WP_075506504; 394 aa, and WP_099339815; 396 aa, respectively) were found to exhibit no detectable activity with L-ornithine or L-lysine (Table 2). In contrast, L-arginine was decarboxylated by the *Pelagibacter* protein with a *K*_m_ of 2.7 ± 0.12 mM, *k*_cat_ of 0.32 ± 0.0010 s^−1^, and *k*_cat_/*K*_m_ of 120 ± 5.5 M^−1^ s^−1^. For the *Fonsibacter* protein, arginine was decarboxylated with a *K*_m_ of 1.3 ± 0.12 mM, *k*_cat_ of 2.2 ± 0.13 s^−1^, and *k*_cat_/*K*_m_ of 1700 ± 91 M^−1^ s^−1^.

**Fig. 2.**
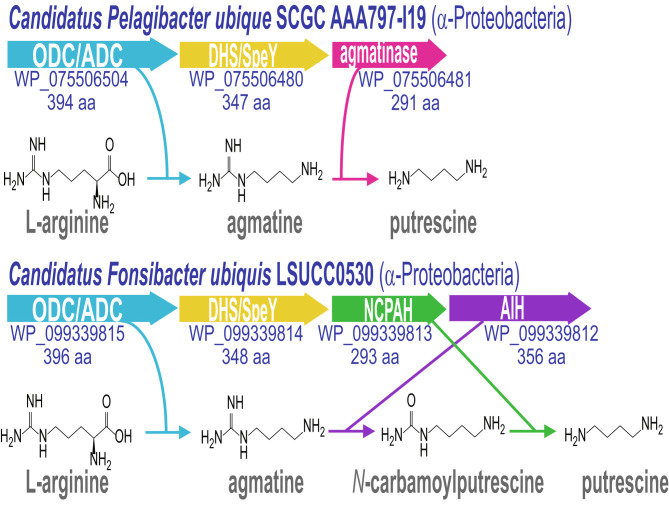
Polyamine biosynthetic gene clusters and pathways in *Ca. Pelagibacter ubique* and *Ca. Fonsibacter ubiquis*. ODC/ADC, an alanine racemase-fold L-ornithine decarboxylase homolog that has evolved to specifically decarboxylate L-arginine; DHS/SpeY, a deoxyhypusine synthase homolog that functions as a homospermidine synthase; NCPAH, *N*-carbamoylputrescine amidohydrolase; AIH, agmatine deiminase/iminohydrolase. GenBank protein accession numbers and encoded protein sizes in amino acids are given below the open reading frames.

The *Pelagibacter* and *Fonsibacter* ODC/ADC ORFs are found in gene clusters encoding homologs of arginase/agmatinase, and AIH and NCPAH, respectively (Figs. 1 and 2), indicating that the phage pyruvoyl-dependent ADCs might increase levels of putrescine using host enzymes to convert agmatine to putrescine. These pelagiphages have acquired pyruvoyl-dependent ADCs that have evolved from AdoMetDC and infect host cells that possess PLP-dependent ADCs that have evolved from ODC, indicating convergent evolution of polyamine biosynthesis encoded by host cell and infecting phage. The only other known example of an AR-fold PLP-dependent ODC that has evolved into an ADC is the enzyme encoded by the chlorovirus PBCV-1 (17). PBCV-1 also encodes AIH and NCPAH (*SI Appendix*, Table S2), suggesting that its host cell does not have sufficient capacity to synthesize putrescine from agmatine. It is likely that the *Pelagibacter* and *Fonsibacter* ODC/ADC enzymes evolved separately from the PBCV-1 ODC/ADC, with the PBCV-1 enzyme being derived from an algal/plant origin (*SI Appendix*, Fig. S2).

AdoMetDC homologs similar to that of the pelagiphage HTVC201P were identified in other uncultured phage genomes, and none of the corresponding ORFs were able to restore spermidine accumulation to *E. coli* BL21Δ*speD* (*SI Appendix*, Figs. S6 and S7). The purified recombinant proteins encoded by a Lake Baikal *Myoviridae* phage (LBP), a freshwater *Myoviridae* phage (UCP01), and three uncultured freshwater *Caudovirales* phages (UCP02, UCP03, and UCP04) were kinetically analyzed (Table 3). LBP, UCP02, and UCP03 AdoMetDC homologs exhibited no detectable decarboxylase activity with L-arginine or L-lysine but were active on L-ornithine with *k*_cat_/*K*_m_ of 240 ± 9.6, 190 ± 14, and 210 ± 4.1 M^−1^ s^−1^, respectively. UCP04 exhibited a trace activity with L-arginine but was active on L-ornithine with a *k*_cat_/*K*_m_ of 170 ± 9.9 M^−1^ s^−1^. UCP01 exhibited no detectable decarboxylase activity with L-lysine but was active with L-arginine (*k*_cat_/*K*_m_ = 66 ± 3.0 M^−1^ s^−1^) and L-ornithine (*k*_cat_/*K*_m_ = 160 ± 4.6 M^−1^ s^−1^). All five AdoMetDC homologs exhibited very similar pyruvoyl-dependent ODC decarboxylase activity. These pyruvoyl-dependent ODCs represent a third protein fold able to decarboxylate L-ornithine in addition to the PLP-dependent AR and aspartate aminotransferase folds.

**Table 3.** Kinetic characterization of AdoMetDC homologs with L-ornithine, L-arginine, and L-lysine

Bacteriophage/enzyme	Substrate	*K*_m_ (mM)	*k*_cat_ (s^1^)	*k*_cat_/*K*_m_ (M^1^s^1^)
Pelagiphage HTVC201P	L-ornithine	n.d.	n.d.	n.d.
Pelagiphage HTVC201P	L-arginine	0.87 ± 0.11	0.25 ± 0.11	290 ± 32
Pelagiphage HTVC201P	L-lysine	n.d.	n.d.	n.d.
Lake Baikal phage, Myoviridae (LBP)	L-ornithine	3.8 ± 0.43	0.92 ± 0.040	240 ± 9.6
Lake Baikal phage, Myoviridae (LBP)	L-arginine	n.d.	n.d.	n.d.
Lake Baikal phage, Myoviridae (LBP)	L-lysine	n.d.	n.d.	n.d.
Uncultured Myoviridae sp. (UCP01)	L-ornithine	5.5 ± 0.29	0.89 ± 0.030	160 ± 4.6
Uncultured Myoviridae sp. (UCP01)	L-arginine	2.2 ± 0.29	0.15 ± 0.010	66 ± 3.0
Uncultured Myoviridae sp. (UCP01)	L-lysine	n.d.	n.d.	n.d.
Uncultured Caudovirales phage (UCP02)	L-ornithine	2.4 ± 0.19	0.45 ± 0.020	190 ± 14
Uncultured Caudovirales phage (UCP02)	L-arginine	n.d.	n.d.	n.d.
Uncultured Caudovirales phage (UCP02)	L-lysine	n.d.	n.d.	n.d.
Uncultured Caudovirales phage (UCP03)	L-ornithine	0.62 ± 0.010	0.13 ± 0.0010	210 ± 4.1
Uncultured Caudovirales phage (UCP03)	L-arginine	n.d.	n.d.	n.d.
Uncultured Caudovirales phage (UCP03)	L-lysine	n.d.	n.d.	n.d.
Uncultured Caudovirales phage (UCP04)	L-ornithine	0.44 ± 0.040	0.080 ± 0.0040	170 ± 9.9
Uncultured Caudovirales phage (UCP04)	L-arginine	2.6 ± 0.81	0.0030 ± 0.0010	1.3 ± 0.22
Uncultured Caudovirales phage (UCP04)	L-lysine	n.d.	n.d.	n.d.

n.d., no detectable activity. All assays performed at 26 °C (±SD of three replicate assays). GenBank protein accession numbers and amino acid sizes: LBP (ATV46410; 119 aa), UCP01 (QMP83838; 117 aa), UCP02 (CAB5221556; 120 aa), UCP03 (CAB4131572; 122 aa), and UCP04 (CAB4162964; 122 aa).

Bacterial and archaeal functionally validated class 1b AdoMetDC proteins were aligned with virus- and phage-encoded bona fide AdoMetDCs, and with virus and phage AdoMetDC homologs, and used to generate a phylogenetic tree (*SI Appendix*, Fig. S8). Three strongly supported clades are evident: The first clade contains only bona fide AdoMetDCs from bacteria, archaea, phage, and eukaryotic viruses, the second contains phage AdoMetDC homologs with ADC activity, and the third contains phage AdoMetDC homologs with ODC activity except for one bona fide AdoMetDC from *Sinorhizobium* phage.

**Fig. 3.**
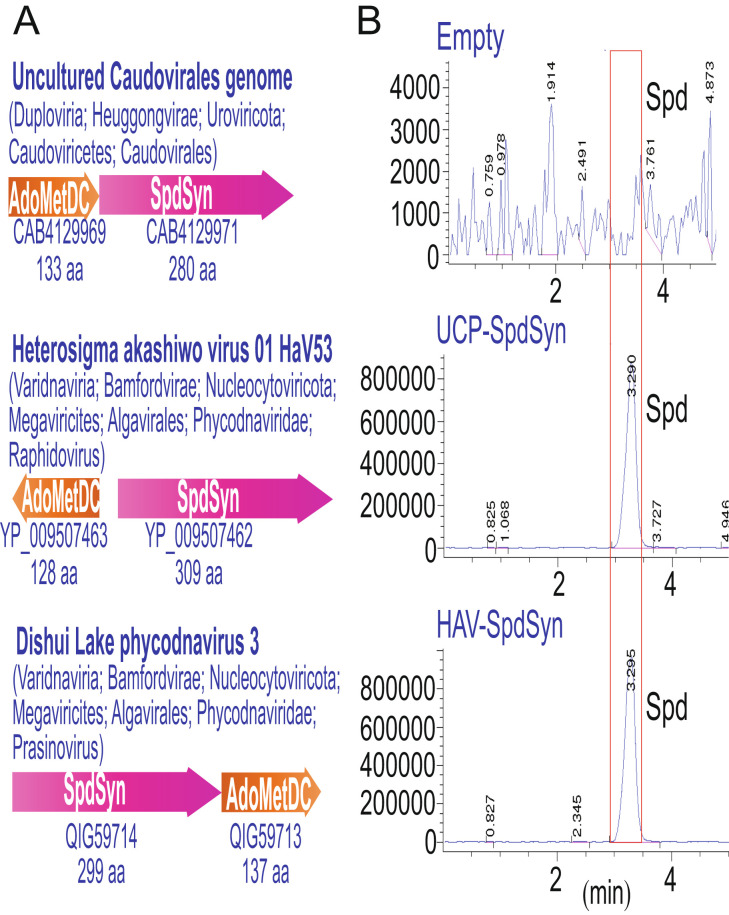
Phage and virus spermidine synthase gene clusters and biochemical function. (*A*), Gene clusters of spermidine synthase (SpdSyn/SpeE) and *S*-adenosylmethionine decarboxylase (AdoMetDC/SpeD). GenBank protein accession numbers and encoded protein sizes in amino acids are given below the open reading frames. (*B*), Extracted ion chromatograms (EICs) from LC–MS analysis of cell extracts of *E. coli* BL21Δ*speE* expressing phage and virus spermidine synthase homologs after induction by 500 µM IPTG. The EICs (457.7/458.7) for tribenzoylated spermidine from cell extracts of *E. coli* BL21Δ*speE* (deficient in spermidine) expressing either empty pETDuet-1, pETDuet-1 with the uncultured Caudovirales genome putative SpdSyn (UCP-SpdSyn), or *H. akashiwo* 01 virus putative SpdSyn (HAV-SpdSyn) are shown. The vertical axis represents arbitrary units of ion intensity.

Spermidine biosynthesis requires the activity not only of AdoMetDC but also of the aminopropyltransferase SpdSyn/SpeE (Fig. 1). We identified one phage and two virus genomes that encode SpdSyn and also a bacterial-type class 1B AdoMetDC, in each case as a clustered gene pair (Fig. 3*A*). The arrangement of the two ORFs is configured differently in each genome. *H. akashiwo* virus HaV53 is a phycodnavirus of 274,792 bp (34) that is known to infect the stramenopile golden brown alga *H. akashiwo* (35). The natural hosts of the freshwater Dishui Lake phycodnavirus 03 (194,169 bp) and uncultured freshwater reservoir *Caudovirales* phage are unknown (36, 37). To assess the biochemical function of the SpdSyn homologs from the *H. akashiwo* virus and uncultured *Caudovirales* phage, each ORF was expressed in a SpdSyn (*speE*) gene deletion mutant of *E. coli* BL21 (Δ*speE*) that completely lacks spermidine when grown in chemically defined medium. Expression of each ORF restored spermidine accumulation in BL21Δ*speE*, confirming that the ORFs encode functional SpdSyn (Fig. 3*B*). The function of the corresponding AdoMetDC homolog ORFs was confirmed by expression in *E. coli* BL21Δ*speD* (*SI Appendix*, Figs. S4–S7), and expression of each AdoMetDC homolog restored spermidine accumulation in BL21*speD*.

BLASTP analysis indicates that the closest homologs of the *H. akashiwo* virus and Dishui Lake phycodnavirus 03 SpdSyn are two, almost identical homologs from the chlorophyte volvocine alga *Tetrabaena socialis* (*SI Appendix*, Fig. S9), which also encodes a homolog of thermospermine synthase (PNH04653; 321 aa), and a eukaryotic-type class 2 AdoMetDC (PNH10116; 383 aa) that is most closely related to AdoMetDCs from other volvocine algae. The volvocine algae include single-celled members like *Chlamydomonas reinhardtii*, the 4-celled *T. socialis*, the 8- or 16-celled *Gonium pectorale*, and the colonial, differentiated multicellular *Volvox* species. A maximum likelihood tree of bacterial, archaeal, eukaryotic, and virus SpdSyn homologs reveals that the *T. socialis* SpdSyn paralogs are the closest relatives of the *H. akashiwo* virus and Dishui Lake phycodnavirus SpdSyns but that they are phylogenetically very distant from the other volvocine algae SpdSyns.

The genome of *T. socialis* contains two giant endogenous virus elements that are essentially integrated copies of large viruses, and the *T. socialis* SpdSyn homologs are derived from those integrated virus elements (38). It is therefore likely that *T. socialis* SpdSyns but not AdoMetDC were horizontally acquired from phycodnaviruses like *H. akashiwo* virus and Dishui Lake phycodnavirus. However, the virus and *T. socialis* SpdSyns are themselves most closely related to bacterial and not eukaryotic SpdSyns (*SI Appendix*, Fig. S9), and the virus AdoMetDCs are also the bacterial-type 1b form. The uncultured *Caudovirales* phage SpdSyn is phylogenetically unrelated to the two virus SpdSyns and is most closely related to homologs from the γ-Proteobacteria (*SI Appendix*, Fig. S9) similar to the corresponding AdoMetDC.

### Virus-Encoded Homospermidine Biosynthesis.

**Fig. 4.**
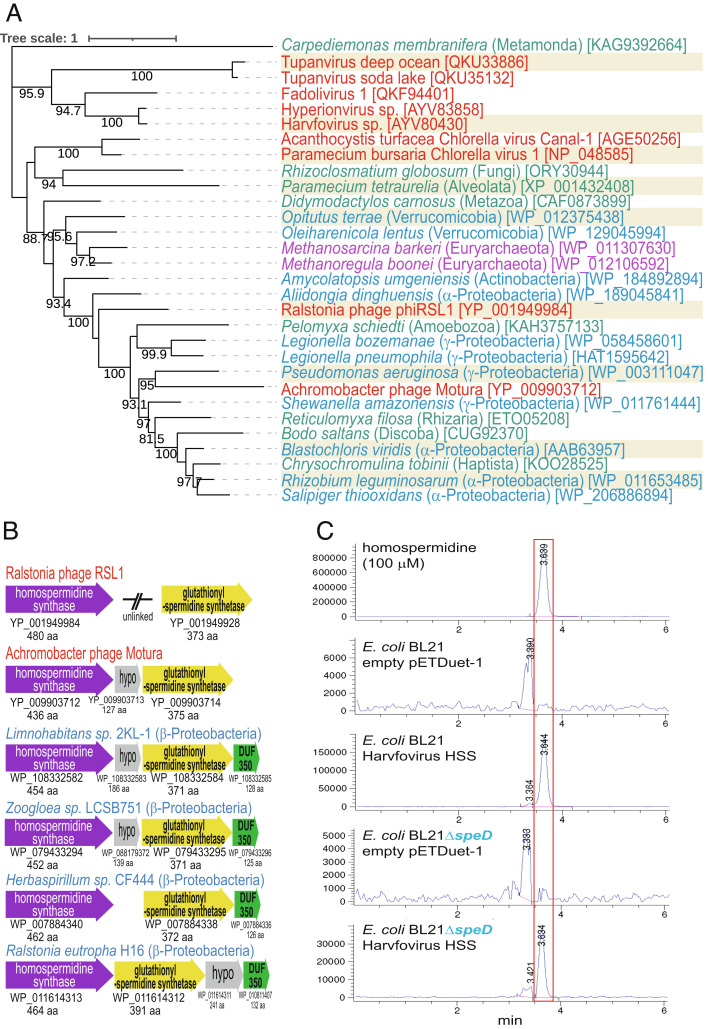
Viral and phage homospermidine synthases. (*A*) Maximum likelihood tree of homospermidine synthases. Species: red, viruses; blue, bacteria; purple, archaea; green, eukaryote. Species names are followed by phyla or class in parentheses, and GenBank protein accession numbers in brackets. Proteins highlighted in tan are biochemically validated. Numerical values represent percentage bootstrap support above 80% from 1,000 ultrafast bootstrap analyses. The scale bar represents the average number of amino substitutions per site. (*B*) Gene clusters containing homospermidine synthase and glutathionylspermidine synthetase: hypo, hypothetical protein; DUF, domain of unknown function. (*C*) LC–MS analysis of cell extracts of *E. coli* BL21 (spermidine replete) and BL21Δ*speD* (deficient in spermidine) expressing the Harvovirus homospermidine synthase homolog (AYV80430; 483 aa) from pETDuet-1. After induction of gene expression by IPTG, cell extracts were benzoylated and subjected to LC–MS analysis. The extracted ion chromatograms for tribenzoylated homospermidine (471.9:472.9) are shown. The vertical axis represents arbitrary units of relative ion intensity. A pure standard of homospermidine was a kind gift of Patrick Woster, Medical University of South Carolina.

Homospermidine is a symmetrical structural analog of spermidine, containing two aminobutyl moieties (Fig. 1), and is not usually found in eukaryotes except in some plants and single-celled eukaryotes (1). An aminobutyl group from homospermidine can be transferred by human DHS to eIF5a (Fig. 1) to form the essential deoxyhypusine modification (39). In the single-celled eukaryote *Paramecium tetraurelia*, homospermidine replaces the essential role of spermidine in growth (40). The mammalian spermidine/spermine *N*-acetyltransferase (SSAT) recognizes only the aminopropyl side of spermidine and does not *N*-acetylate homospermidine (41), allowing homospermidine to evade SSAT-induced catabolism. In bacteria, homospermidine is relatively widespread and is essential for normal growth in the α-proteobacterium *Rhizobium leguminosarum* (20). Homospermidine can be synthesized from putrescine in bacteria by two different nonhomologous, isofunctional enzymes: a DHS-like homolog SpeY, which is not involved in deoxyhypusine formation (20, 42), and homospermidine synthase (HSS), which is related to carboxyspermidine dehydrogenase and lysine 6-dehydrogenase (20, 43, 44). We were unable to detect any SpeY homologs encoded in virus or phage genomes by BLASTP. However, a functional homolog of HSS was previously reported and encoded by chlorovirus PBCV-1 (16) and is present in at least 40 sequenced chlorovirus genomes (*SI Appendix*, Table S2). A functional HSS is encoded in *Ralstonia solanacearum* phage ϕRSL1 (20, 45). We have now identified HSS homologs in the *Imitervirales* viruses, Tupanvirus soda lake, Tupanvirus deep ocean, Harvovirus, Hyperionvirus, and Fadolivirus 1, and in *Achromobacter* phage Motura (Fig. 4*A*).

The closest homologs of the *Ralstonia* phage ϕRSL1 and *Achromobacter* phage Motura HSS proteins are found in the γ- and α-Proteobacteria and diverse single-celled eukaryotes that have likely acquired *hss* genes from bacteria. The *Imitervirales* HSS sequences are most similar to each other and are excluded from bacteria and archaea in a phylogenetic tree (Fig. 4*A*). In the *Achromobacter* phage Motura genome, *hss* is physically clustered with a homolog of glutathionylspermidine synthetase (*gss*), whereas in the ϕRSL1 genome, *hss* and *gss* are unlinked (Fig. 4*B*). The physical clustering of *hss* and *gss* is found in a number of β-Proteobacteria genomes and indicates the potential for formation of glutathionylhomospermidine. To confirm the enzymatic activity of the virus HSS homologs, we selected for functional analysis of Harvovirus HSS (AYV80430; 483 aa) and Tupanvirus soda lake HSS (QKU35132; 420 aa) that exhibit only 29% amino acid identity to each other. The ORFs encoding each protein were expressed in either *E. coli* BL21, which contains putrescine and spermidine, or *E. coli* BL21Δ*speD*, which lacks spermidine. Homospermidine was identified in benzoylated cell extracts by LC–MS detection of tribenzoylated homospermidine. The identity of homospermidine produced by Harvovirus HSS was further confirmed by comparison with a pure sample (Fig. 4*C*) and for the Tupanvirus soda lake HSS with the product of the bona fide HSS from *Agrobacterium tumefaciens* (*SI Appendix*, Fig. S10). Expression of the Harvovirus HSS and the Tupanvirus soda lake HSS (Fig. 4*C* and *SI Appendix*, Fig. S10) produced homospermidine in both *E. coli* strains in the presence (BL21) and absence (BL21Δ*speD*) of native spermidine. A possible advantage for eukaryotic viruses in producing homospermidine rather than spermidine could be avoidance of host-mediated cytoplasmic *N*-acetylation that would otherwise eliminate functionality of host- and virus-produced spermidine.

### Virus-Encoded Spermidine *N*-Acetylation and Deacetylation.

When spermidine levels are in excess, bacterial cells employ the enzyme spermidine *N*-acetyltransferase (SAT) to *N*-acetylate spermidine, thereby abrogating spermidine functionality by forming *N*-acetylspermidine. As SAT is a key regulator of spermidine function, we searched by BLASTP and PSI-BLAST for homologs encoded in phage and virus genomes using sequences of characterized SATs: human SSAT, *E. coli* SpeG, *B. subtilis* BltD, and *Enterococcus faecalis Ef*BltD (46–49). All strains of chlorovirus genomes encode a homolog of the PCBV-1 SAT, although the amino acid sequences of some chlorovirus SATs conserve only 51% identity to the PCBV-1 SAT (*SI Appendix*, Table S2). Conservatively, we identified nine candidate SATs outside of the chloroviruses (*SI Appendix*, Table S4).

To assess SAT activity, each protein was expressed in an SAT (Δ*speG*) knockout strain of *E. coli* BL21, and benzoylated cell extracts were analyzed by LC–MS to detect dibenzoylated *N*-acetylspermidine (*SI Appendix*, Figs. S11 and S12). Peaks for dibenzoylated *N*-acetylspermidine (m/z = 396.2) and its sodium adduct (m/z = 418.2) confirmed SAT activity of seven phage and one virus proteins. In the *E. coli* BL21Δ*speG* strain lacking SAT activity, a small extracted ion chromatogram peak was present at 3.28 min, whereas the pure *N*-acetylspermidine peak and the parental strain *N*-acetylspermidine peak eluted at 3.36/3.33 min. The highest level of *N*-acetylspermidine accumulation was obtained with the plasmid-borne *E. coli speG* gene. The other SATs that caused an accumulation of *N*-acetylspermidine were derived from *Erwinia* phages vB_EamM_Caitlin and phiEaH2, *Bacillus* phage SPBc2, and *Streptococcus* phage phi-m46.1 (*SI Appendix*, Fig. S11) and from *Klebsiella* phage ST13-OXA48phi12.4, *Lactococcus* phage bIL311, *Escherichia* phage vB_EcoM-Goslar, and the eukaryotic virus Pithovirus LCPAC302 (Pithoviridae) (*SI Appendix*, Fig. S12). Expression of the candidate SAT ORF from *Salmonella* phage SPN3US (YP_009153389; 154 aa) did not result in *N*-acetylspermidine accumulation.

Five of the seven phage proteins exhibiting SAT activity display 100% amino acid sequence identity to proteins encoded in bacterial genomes. Only the *Erwinia* phage SAT proteins are distinct from any bacterial proteins. Of the other phages that encode SAT proteins with complete amino acid identity to bacterial proteins, the *Lactococcus* phage bIL311 and *Streptococcus* phage phi-m46 are known to be temperate phages that exist as integrated prophages (50, 51). The bacterial SATs with 100% identity to the phage SATs are therefore likely to be encoded by integrated prophages. In contrast, the *Erwinia* phages are known to be strongly lytic and do not integrate into the host genome (52). The closest bacterial homologs of the *Erwinia* phage SATs display only 73% amino acid identity to the phage SATs.

It appears paradoxical that some phages should encode SAT since spermidine is required for phage T4 DNA synthesis and phage maturation in *E. coli* (53). However, in vitro, spermidine is necessary to avoid nonspecific integration of new CRISPR protospacers catalyzed by the *E. coli* Cas1–Cas2 integrase, and spermidine significantly prevents off-target integration (54). These in vitro data suggest that spermidine may be essential for the correct functioning and efficiency of the CRISPR machinery in vivo. Furthermore, spermidine is also required in vitro for maintaining the accuracy of type II restriction endonucleases (55). During the early stages of infection, the host cell depends on CRISPR systems and restriction endonucleases to eliminate phage DNA; therefore, if the phage SATs were expressed early after infection, temporary depletion of spermidine would allow the phage to establish a more robust infection until spermidine became necessary for translation of the phage RNA.

In eukaryotic cells, spermidine is required by RNA and DNA viruses for replication (22), and this suggests that reducing host spermidine levels during virus infection could be a host response to limit virus replication. Indeed, interferon-induced expression of SSAT in a human cell line was shown to be correlated with restriction of Zika and chikungunya viruses (56). Acetylation of host spermidine would sequester it into the inactive form *N*-acetylspermidine. A corollary of this finding is that viruses might increase polyamine levels by encoding their own polyamine biosynthetic enzymes, or alternatively, host spermidine could be released from inactive *N*-acetylspermidine via the activity of virus-encoded amidohydrolases. We used the amino acid sequence of the previously characterized acetylpolyamine amidohydrolase (APAH) from the α-proteobacterium *Mycoplana ramosa* (57) to search for virus-encoded homologs and conservatively identified ten virus APAH homologs, all from the *Imitervirales* (*SI Appendix*, Table S5). Candidate APAH proteins from Klosneuvirus KNV1 (ARF11327), Tupanvirus soda lake (QKU35181), and Hyperionvirus sp. (AYV83810) were selected for expression in *E. coli*, purification, and kinetic analysis of substrate specificity.

In mammalian cells, SSAT acetylates only the aminopropyl side of spermidine to form *N*^1^-acetylspermidine (structure shown in *SI Appendix*, Fig. S13), whereas the histone acetyltransferase P300/CBP-associated factor (P/CAF) acetylates the aminobutyl side to form *N*^8^-acetylspermidine, which has also been identified in *Acanthamoeba culbertsoni* (58). The potential virus APAH proteins were assayed with *N*^1^- and *N*^8^-acetylspermidine and with *N*-acetylputrescine, and the *M. ramosa* bacterial APAH was used as a positive comparative control (Table 4). APAH activity was followed by colorimetrically measuring the release of acetate from the substrates (*SI Appendix*, Fig. S13). Relative efficiency (*k*_cat_/*K*_m_) of the APAH enzymes with *N*^1^-acetylspermidine, *N*^8^-acetylspermidine, and *N*-acetylputrescine was found to be as follows: *M. ramosa* (100/25/91), Tupanvirus soda lake (100/120/29), Klosneuvirus KNV1 (100/81/10), and Hyperionvirus sp. (100/25/0.2). The three virus APAH proteins preferred *N*^1^- and *N*^8^-acetylspermidine to *N*-acetylputrescine, whereas the bacterial enzyme exhibited almost equal preference for *N*^1^-acetylspermidine and *N*-acetylputrescine.

**Table 4.** Kinetic parameters for *N*-acetylpolyamine amidohydrolases

Bacterium/virus protein	Substrate	*K_M_*(mM)	*k*_cat_ (s^1^)	*k*_cat_/*K_M_*(s^1^ M^1^)
*M. ramosa*	*N*^1^-acetylspermidine	0.63 ± 0.040	5.8 ± 0.14	9,300 ± 450
*M. ramosa*	*N*^8^-acetylspermidine	2.5 ± 0.71	5.6 ± 1.1	2,300 ± 190
*M. ramosa*	*N*-acetylputrescine	0.55 ± 0.010	4.7 ± 0.080	8,500 ± 40
Tupanvirus soda lake	*N*^1^-acetylspermidine	0.24 ± 0.030	2.9 ± 0.040	12,630 ± 1800
Tupanvirus soda lake	*N*^8^-acetylspermidine	0.35 ± 0.03	5.3 ± 0.65	15,150 ± 550
Tupanvirus soda lake	*N*-acetylputrescine	1.1 ± 0.13	3.9 ± 0.10	3,700 ± 120
Klosneuvirus KNV1	*N*^1^-acetylspermidine	0.24 ± 0.020	5.5 ± 0.41	23,000 ± 1400
Klosneuvirus KNV1	*N*^8^-acetylspermidine	0.45 ± 0.010	8.3 ± 0.25	19,000 ± 270
Klosneuvirus KNV1	*N*-acetylputrescine	3.2 ± 0.10	7.0 ± 0.090	2,200 ± 71
Hyperionvirus sp.	*N*^1^-acetylspermidine	1.6 ± 0.16	37 ± 1.6	24,000 ± 1900
Hyperionvirus sp.	*N*^8^-acetylspermidine	5.0 ± 0.73	30 ± 4.4	6,100 ± 470
Hyperionvirus sp.	*N*-acetylputrescine	27 ±2.7	1.0 ± 0.080	38 ± 0.93

*M. ramosa* (BAA01256; 341 aa), Tupanvirus soda lake (QKU35181; 354 aa), Klosneuvirus KNV1 (ARF11327; 345 aa), and Hyperionvirus sp. (AYV83810; 351 aa). Enzymes assayed at 26 °C (±SD of three replicate assays).

To cross-validate the substrate preferences of the virus APAH enzymes, the Tupanvirus soda lake (59) protein was incubated with *N*^1^-acetylspermidine or *N*-acetylputrescine, and the released polyamine was detected by LC–MS after benzoylation (*SI Appendix*, Fig. S14). A confounding factor was that the acetylated polyamine stocks contained a background level of free polyamine, but the virus APAH caused the release of 10-fold more spermidine and fivefold more putrescine from the corresponding acetylated polyamines, which is similar to the ratio detected by acetate release.

**Fig. 5.**
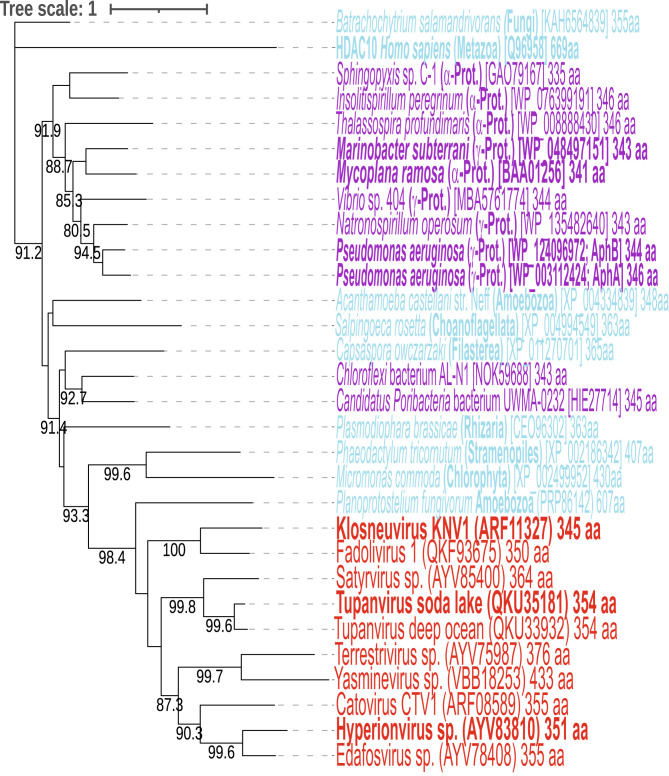
Maximum likelihood phylogenetic tree of acetylpolyamine amidohydrolases and homologs. Biochemically confirmed APAH proteins are indicated in bold. Red, virus proteins; blue, eukaryote; purple, bacteria. Species names are followed by phyla or class in parentheses, and GenBank protein accession numbers are shown in brackets followed by protein size in amino acids. Numerical values represent percentage support above 80% after 1,000 ultrafast bootstraps. The scale bar represents the average number of amino substitutions per site.

The functional demonstration of APAH activity by the three virus proteins indicates that the other virus homologs are also likely to be *N*-acetylspermidine amidohydrolases, releasing spermidine from *N*^1^- and *N*^8^-acetylspermidine. The ability of the virus *N*-acetylspermidine amidohydrolases to act on both *N*^1^- and *N*^8^-acetylspermidine is in contrast to the vertebrate histone deacetylase HDAC10 enzyme that deacetylates only *N*^8^-acetylspermidine (60). An *N*^1^-acetylspermidine deacetylase has yet to be identified in eukaryotes. We constructed a phylogenetic tree of the virus homologs, four biochemically characterized APAH enzymes from bacteria [*M. ramosa*, *Marinobacter subterrani* (61), and *P. aeruginosa* (62)], and other similar proteins (Fig. 5). The virus APAH homologs are most similar to each other and to the amoebozoan eukaryote *Planoprotostelium fungivorum* in a separate and robustly supported clade. This could suggest that the different virus APAH proteins have a common origin, ultimately from bacteria via an amoeba.

### Virus-Encoded eIF5a.

The only known essential function of spermidine conserved across eukaryotes is the hypusine modification of a translation factor eIF5a (9). DHS transfers the aminobutyl group of spermidine to a lysine residue in eIF5a to form deoxyhypusine, which is then hydroxylated to form hypusinated eIF5a. Homospermidine is compatible with eIF5a hypusination due to the presence of aminobutyl groups. Kaposi’s sarcoma-associated herpesvirus (KSHV) up-regulates host ODC and DHS during its latent phase, resulting in increased levels of host hypusinated eIF5a, and critical KSHV proteins require host hypusinated eIF5a for synthesis (63). Ebola virus (EBOV) requires host hypusinated eIF5a for virus posttranscriptional expression and in addition requires host polyamines for EBOV polymerase-synthesized mRNA (12).

We screened virus proteins by BLASTP for homologs of the eIF5a-modifying enzyme DHS, but none were detected. In contrast, we detected eIF5a homologs encoded in five *Imitervirales* and two *Algavirales* viruses (*SI Appendix*, Table S6). No eIF5a homolog was detected among phage proteins. Although an eIF5a homolog is present in Tupanvirus deep ocean, the Tupanvirus soda lake genome homolog contains a frameshifting mutation and is unlikely to be active. It is notable that every *Imitervirales* virus that encodes eIF5a also encodes polyamine biosynthetic enzymes unlike the *Algavirales* genomes.

### Pathways and Metabolic Modules.

**Fig. 6.**
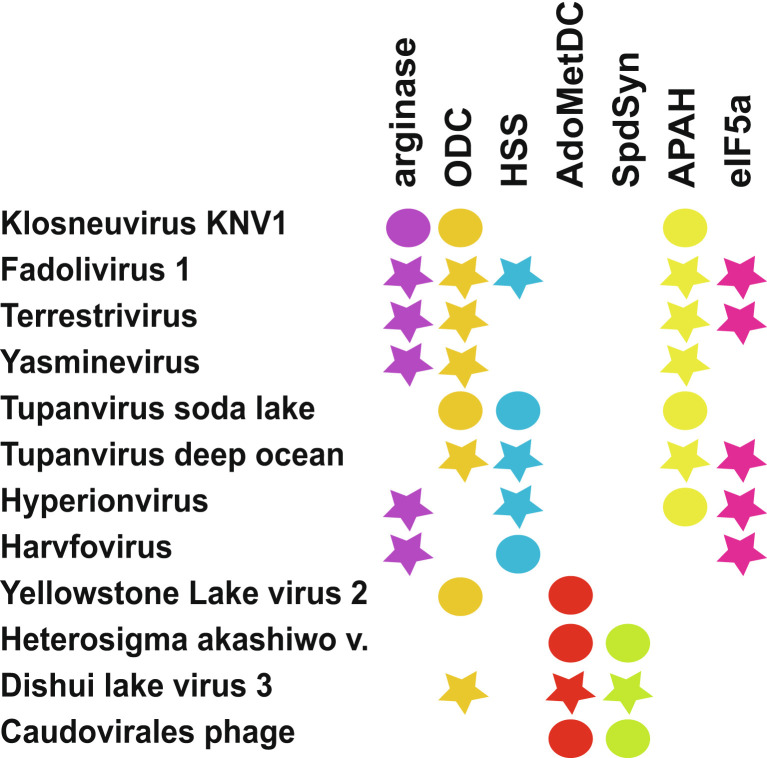
Viruses and a phage encoding more than one polyamine metabolism-related enzyme. Filled circles indicate enzymes biochemically characterized in this study; stars represent uncharacterized homologs. ODC, L-ornithine decarboxylase; HSS, homospermidine synthase; AdoMetDC, S-adenosylmethionine decarboxylase (SpeD); SpdSyn, spermidine synthase (SpeE); APAH, acetylpolyamine amidohydrolase; eIF5a, translation factor 5a.

The presence of more than a single polyamine metabolic enzyme encoded in virus genomes is found primarily in eukaryotic viruses, although one phage genome we identified encodes AdoMetDC and SpdSyn, constituting a putrescine to spermidine biosynthetic module (Fig. 6). By far the most commonly found polyamine metabolic enzyme encoded in phage genomes is AdoMetDC, a rate-limiting step in polyamine biosynthesis, which has the advantage of also being a small, PLP-independent protein.

Two distinct polyamine metabolic pathways can be distinguished between the *Imitervirales* and *Algavirales* genomes that encode polyamine metabolic enzymes. The *Imitervirales* encode either a partial or complete pathway from arginine to homospermidine via arginase, ODC, and HSS. They may also encode an APAH enzyme to release spermidine from sequestered *N*-acetylspermidine and may encode eIF5a. We characterized the L-arginine to putrescine route encoded by Klosneuvirus KNV1 (arginase and ODC), the L-ornithine to homospermidine route in Tupanvirus soda lake (ODC and HSS), and the *N*-acetylspermidine to spermidine route in both Klosneuvirus KNV1 and Tupanvirus soda lake (APAH). Single metabolic enzymes were characterized from Harvovirus (HSS) and Hyperionvirus (APAH). The most complete representation of polyamine metabolism is encoded in Fadolivirus 1, which encodes homologs of arginase, ODC, HSS, APAH, and eIF5a. In principle, Fadolivirus can reprogram host metabolism to convert L-arginine to deoxyhypusinated eIF5a (using host DHS) via L-ornithine, putrescine, and homospermidine or from *N*-acetylspermidine via spermidine. The *Algavirales* encode a complete or partial route from L-ornithine to spermidine (ODC, AdoMetDC, and SpdSyn) but would not be able to release spermidine from *N*-acetylspermidine. Presumably, the *Imitervirales* genomes that do not encode HSS but which do encode the ability to produce putrescine must then rely on host cell AdoMetDC and SpdSyn to produce spermidine rather than homospermidine. It is intriguing that both the *Imitervirales* and *Algavirales* genomes encode bacterially derived polyamine metabolic pathways, particularly HSS and class 1b AdoMetDC.

## Conclusions

Bacterial and eukaryotic viruses often encode auxiliary metabolic enzymes that subvert host metabolism to enable viral replication (64). The auxiliary polyamine metabolism encoded across the virosphere is extensive. It includes arginase to convert L-arginine to L-ornithine, PLP- and pyruvoyl-dependent ODCs to convert L-ornithine to putrescine, PLP- and pyruvoyl-dependent ADCs to convert L-arginine to agmatine, and AIH and NCAPH to convert agmatine to putrescine. Putrescine can be converted to spermidine by AdoMetDC and SpdSyn, and HSS converts putrescine to homospermidine. APAH converts *N*-acetylspermidine to spermidine, and SAT converts spermidine to *N*-acetylspermidine. Thus, in the virosphere, there are alternative ODC and ADC pathways to putrescine, alternative forms of ODC or ADC, alternative solutions to polyamine biosynthesis (spermidine or homospermidine), an alternative to spermidine biosynthesis, i.e., the release of spermidine from host *N*-acetylspermidine, and a way to control free spermidine levels through *N*-acetylation. Together, these different virus-encoded auxiliary metabolic strategies for spermidine control illuminate its prominent role in the virus replication cycle and by inference indicate the central role of spermidine in the core physiological processes of the host cell.

Why would giant viruses be more likely to encode polyamine metabolic enzymes? It is known that they encode more translation-related genes (65), suggesting that the host translational machinery might be insufficient for the protein synthetic requirements of the virus. Consistent with this would be the possibility of insufficient host spermidine to posttranslationally modify the essential translation factor eIF5a, requiring therefore virus-encoded spermidine or homospermidine biosynthesis.

The bacteriophage-encoded AdoMetDC homologs that have been neofunctionalized as pyruvoyl-dependent ODCs evolved either as virus-encoded genes or were acquired as neofunctionalized AdoMetDCs from ancestral bacterial hosts. Pyruvoyl-dependent ODCs have not been reported in bacteria or any other organism, and we are currently seeking such enzymes in bacteria and archaea.

## Materials and Methods

Detailed descriptions of materials and experimental protocols can be found in *SI Appendix*. These include protein expression and purification, enzyme assays and reactions, mass spectrometry, and phylogenetic analysis.

## Supplementary Material

Appendix 01 (PDF)

## Data Availability

All study data are included in the article and/or *SI Appendix*.
