# Supplementary material for: Functional polyamine metabolic enzymes and pathways encoded by the virosphere
Source: Proc Natl Acad Sci U S A. 2023 Feb 21;120(9):e2214165120. doi: 10.1073/pnas.2214165120 (PMC9992855; doi:10.1073/pnas.2214165120)
Supplement: Supplementary file 1 — Appendix 01 (PDF) [file pnas.2214165120.sapp.pdf]

**Supplementary Information**

# Functional polyamine metabolic enzymes and pathways encoded by the virosphere

Bin Li[a], Jue Liang[a], Hamid R. Baniasadi[a], Margaret A. Phillips[a] and Anthony J Michael[a,1]

[a]Department of Biochemistry, University of Texas Southwestern Medical Center, Dallas, TX 75214

**Material and Methods**

**Supplementary Tables**

**Supplementary Figures**

## Supplementary Experimental Section

### Bacteriophage, virus and bacterial genes

All genes tested were synthesized by GenScript with *E. coli*-optimized codons. Amino acid sequences were obtained from GenBank.

### Gene expression in *E. coli* BL21 (DE3) strains

Gene sequences corresponding to complete open reading frames were cloned into pETDuet-1 (Novogene) and transformed into *E. coli* BL21 strains. For some of the phage and virus AdoMetDC/*speD* genes, the *E. coli* SpdSyn/*speE* ORF was co-expressed from the same pETDuet-1 plasmid. The BL21Δ*speD* and BL21Δ*speE* strains were described previously (1, 2). Transformed strains were grown twice in 2 ml M9 polyamine-free medium at 37 °C overnight. A 1.0 ml aliquot of the culture was then centrifuged, the supernatant discarded, and cells resuspended in 10 ml M9 medium and grown at 37 °C to $OD_{600}$ = 0.5. Gene expression from pETDuet-1 was induced by addition of 0.2 mM isopropyl-β-d-thiogalactopyranoside (IPTG) and the culture was maintained at 16 °C, overnight. Cells were then centrifuged, and polyamines extracted.

### Protein overexpression and purification

Open reading frames were expressed from pET28 plasmids (Novogene) in *E. coli* BL21 (DE3). Cells containing expression plasmids were grown in LB medium to mid-log phase at 37 °C with aeration before addition of 0.2 mM IPTG, and then cells were maintained at 16 °C overnight. Proteins were purified as described previously (3). Protein purity was assessed using sodium dodecyl sulfate-polyacrylamide gel electrophoresis, and protein concentration was determined using a Biotek Synergy Multi-Mode Microplate reader at $OD_{280}$ employing the molecular weight and protein extinction coefficients program. The overnight culture was then centrifuged at 20,000 rpm (48,400 x *g*) in a Beckman JA25.5 rotor, and resuspended in buffer A (see below) containing protease inhibitor cocktail and lysed in a cell disruptor at 10,000 p.s.i., the lysate was then centrifuged for 60 min to remove unbroken cells, debris and other insoluble material. The soluble sample was then applied to a 5 ml Hi-Trap chelating HP column (GE Healthcare) that was equilibrated with $NiSO_4$ and buffer A, and the 6 x his-tagged proteins eluted from the column using a gradient of 0-50% buffer B (see below) over 20 column volumes. Protein desalting was achieved by overnight dialysis with dialysis buffer described below. L-ornithine decarboxylase purification buffer A contained 50 mM $K_2HPO_4$ (pH 8.0), 300 mM NaCl, 5 mM imidazole, 1 mM TCEP (disulfide reducing agent), 5% glycerol, and buffer B contained 50 mM $K_2HPO_4$ (pH 8.0), 300 mM NaCl, 500 mM imidazole, 1mM TCEP, 5% glycerol. The dialysis buffer contained 50 mM $K_2HPO_4$ (pH 8.0), 300 mM NaCl, 1mM TCEP, 5% glycerol. Arginase purification buffer A contained 20 mM Tris-HCl buffer (pH 7.5), 500 mM NaCl, 10 mM imidazole, 0.02% Brij35, and Buffer B contained 20 mM Tris-HCl buffer (pH 7.5), 500 mM NaCl, 500 mM imidazole, 0.02% Brij35. The dialysis buffer contained 20 mM Tris-HCl buffer (pH 7.5), 500 mM NaCl, 0.02% Brij35. Acetylpolyamine amidohydrolase lysis buffer contained 50 mM $K_2HPO_4$ (pH 8.0), 300 mM NaCl, 10 mM $MgCl_2$, 10% glycerol, and protease inhibitor cocktail (ThermoFischer Scientific). Buffer A contained 50 mM $K_2HPO_4$ (pH 8.0), 300 mM NaCl, 1 mM TCEP, 5% glycerol, and buffer B contained 50 mM $K_2HPO_4$ (pH 8.0), 300 mM NaCl, 1 mM TCEP, 500 mM imidazole, 5% glycerol. The dialysis buffer contained 50 mM $K_2HPO_4$ (pH 8.0), 300 mM NaCl, 1 mM DTT and 5% glycerol.

**Decarboxylase assay**

All reactions were performed in 200 µl volumes, with 50 mM HEPES pH 7.7, 100 mM NaCl and 1 mM dithiothreitol, with 0-10 mM L-amino acid. PLP-dependent ODC was assayed with 0.2 mM PLP included. After addition of enzyme, release of $CO_2$ was detected by addition of 100 µl $CO_2$ detection solution (Infinity Carbon Dioxide liquid stable reagent, Thermo Scientific). The decarboxylase reaction was followed using a Biotek plate reader to monitor reaction kinetics, set as: primary wavelength $OD_{340}$, 27 °C, 10 s/read, end reading at 20 min. Kinetic parameters for all enzyme assays ($K_M$, $V_{max}$ and $k_{cat}$) and were calculated in GraphPad Prism using the Michaelis Menten model.

**Arginase/agmatinase assay**

Arginase/agmatinase activity was assayed by colorimetric detection of released urea, employing a urea assay kit (Sigma-Aldrich). Ten µM arginase or agmatinase was mixed into a buffer containing 20 mM Tris-HCl (pH 7.5), 100 µM $MnCl_2$, 200 mM NaCl, 1 mM DTT, and 0-15 mM arginine or agmatine in a total volume of 200 µl. Reactions were incubated at 37 °C for 1 h, 3-5 µl of each reaction was mixed with 42 µl urea assay buffer, 2 µl peroxidase substrate, 2 µl enzyme mix, 2 µl developer, 2 µl converting enzyme, and urea assay buffer was added to a final volume of 100 µl, then covered and incubated at 37 °C for 0-60 min. Each sample was assayed at least three times. Urea production was detected by measuring absorbance at 570 nm. The concentration of urea produced was calculated by comparison with a urea standard curve.

**Acetylpolyamine amidohydrolase (APAH) assay**

Reactions were performed in a total volume of 100 µl containing 50 µl acetate detection kit reaction mixture (Sigma-Aldrich), prepared as described in the kit protocol: 42 µl of acetate assay buffer, 2 µl of acetate enzyme mix, 2 µl of ATP solution, 2 µl of acetate substrate mix and 2 µl of probe. To this was added a 50 µl mixture of 1-3 µM APAH enzyme, and arginine or agmatine concentrations ranging from 0 to 2000 µM in a final concentration of buffer containing 100 mM HEPES (pH 7.4), 10 mM KCl, 4 mM $CaCl_2$, 4 mM $MgCl_2$, and 280 mM NaCl. The reaction was immediately placed in a plate reader and monitored at 450 nm at 26 °C. Measurements were taken every 10 s for 40 min with continuous shaking.

**Polyamine extraction from *E. coli* cells**

Cultures of *E. coli* BL21 strains that had been induced to express polyamine metabolic genes from pETDuet-1 were pelleted by centrifugation, and washed three times by resuspension in PBS. Repelleted cells were resuspended in 200 µl of lysis buffer (100 mM MOPS pH 8.0, 50 mM NaCl, 20 mM $MgCl_2$), frozen in liquid nitrogen, thawed at 37 °C, and this was repeated three times. Sixty µl of 40% trichloroacetic acid was added, mixed well and kept on ice for 5 min. Cellular debris was pelleted by centrifugation at 4°C and the supernatant transferred to a new tube for benzoylation.

**Polyamine benzoylation**

The benzoylation of polyamines improves chromatographic separation and detection. Putrescine, agmatine and *N*-acetylspermidine are benzoylated on two positions, and spermidine on three. To 200 µl of the cell supernatant containing extracted polyamines, 1 ml of 2 M NaOH was added, and then 10 µl of benzoyl chloride, and this mixture was vigorously vortexed for 2 min, and left at room temperature for 1 h. Two ml of saturated NaCl was added, followed by further vortexing for 2 min, then 2 ml of diethyl ether added, vortexing for another 2 min and left at room temperature for 30 min. The upper layer of

diethyl ether containing the polyamines was transferred to a new tube and kept in a chemical hood until fully evaporated.

**Arginase reaction for LC-MS analysis**

The reaction was performed at 37 °C for 60 min in a total volume of 200 µl containing 20 mM Tris-HCl (pH 7.5), 100 µM $MnCl_2$, 200 mM NaCl, 1 mM DTT, 5 mM arginine or agmatine, 10 µM Klosneuvirus KNV1 arginase. The reaction was stopped by heating to 85 °C for 10 min. The boiled enzyme control was heated at 100 °C for one hour.

**Liquid Chromatography-Mass Spectrometry (LC-MS) analysis**

Samples were analyzed on an Agilent 1290 Infinity HPLC system using an Eclipse XDB-C18 column (4.6 x 150 mm, 5 µm particle size) that was coupled to an Agilent 6130 quadrapole ESI mass spectrometer run in positive mode with a scan range of 100 to 1100 m/z. Liquid chromatography was carried out at a flow rate of 0.5 ml/min at 20 °C with a 5 µl injection volume, using a gradient elution with aqueous acetonitrile containing 0.1% formic acid. The gradient was adjusted based on the different polarities of analyzed compounds.

**LC-MS/MS analysis**

LC-MS/MS analysis was conducted using a SCIEX QTRAP 6500+ mass spectrometer coupled to a Shimadzu high-performance liquid chromatography (HPLC) system (Nexera X2 LC-30AD). The ESI source was used in the positive ion mode with the ion spray needle voltage set at 4800 V. Reverse-phase chromatography was performed using a C18 column, ACE 3 C18-PFP 150 x 4.6 mm and 3 µm particle size (Advanced Chromatography Technologies Ltd, Aberdeen, Scotland). The column temperature, sample injection volume and the flow rate were set to 30 °C, 5 µl, and 0.5 ml/min respectively. HPLC solvent and gradient conditions were as follows: solvent A: 0.2% formic acid in water (LC-MS grade) and solvent B: acetonitrile (LC-MS grade). Gradient conditions were: 0-2 min at 95% (A) and 5% (B); 2–5 min, increasing solvent B to 90%; 5-16 min 90% (B); 17-22 min at 95% (A) and 5% (B). Samples were maintained in an autosampler at 4 °C. Analyst 1.7.1 software (Sciex) was used for the data processing. Total run time: 22 min. Flow was diverted to waste for the first 0.5 min and after 18 min.

**Phylogenetic analysis**

All virus and phage proteins were conservatively identified by BLASTP or PSI-BLAST against the non-redundant (NR) protein sequences database of GenBank, using the search term “viruses”. All searches were repeated in June 2022. Protein sequences were aligned with MUSCLE (4), and Maximum Likelihood trees constructed with IQ-TREE (5) at http://iqtree.cibiv.univie.ac.at/ using default parameters for protein sequences and 1000 ultrafast bootstrap analysis (6), with the automatic substitution model. All phylogenetic trees were constructed in the first six months of 2022. Trees were visualized with iTOL (7) and exported to Adobe Illustrator as a .eps file for annotation.

**References**


1. B. Li, *et al.*, Functional Identification of Putrescine C- and N-Hydroxylases. *ACS Chem. Biol.* **11**, 2782-2789 (2016).
2. B. Li, S. Kurihara, S. H. Kim, J. Liang, A. J. Michael, A polyamine-independent role for S-adenosylmethionine decarboxylase. *Biochem. J.* **476**, 2579-2594 (2019).

3. B. Li, J. Liang, C. C. Hanfrey, M. A. Phillips, A. J. Michael, Discovery of ancestral L-ornithine and L-lysine decarboxylases reveals parallel, pseudoconvergent evolution of polyamine biosynthesis. *J. Biol. Chem.* **297**, 101219 (2021).
4. R. C. Edgar, MUSCLE: multiple sequence alignment with high accuracy and high throughput. *Nucleic Acids Re.s* **32**, 1792-1797 (2004).
5. J. Trifinopoulos, L. T. Nguyen, A. von Haeseler, B. Q. Minh, W-IQ-TREE: a fast online phylogenetic tool for maximum likelihood analysis. *Nucleic Acids Res.* **44**, W232-235 (2016).
6. B. Q. Minh, M. A. Nguyen, A. von Haeseler, Ultrafast approximation for phylogenetic bootstrap. *Mol. Biol. Evol.* **30**, 1188-1195 (2013).
7. I. Letunic, P. Bork, Interactive Tree Of Life (iTOL) v5: an online tool for phylogenetic tree display and annotation. *Nucleic Acids Res.* **49**, W293-W296 (2021).

**Table S1. Viruses encoding alanine racemase-fold L-ornithine decarboxylase (ODC) homologues.**

| Virus | Order/ Family | Encoded Protein | Size (a.a.) | % i.d. |
|---|---|---|---|---|
| Bovine gammaherpesvirus 6 | H | YP_009041983 | 431 | 45 |
| *Acanthocystis turfacea* Chlorella virus NE-JV-2 | A | AGE56788 | 371 | 36 |
| ***Paramecium bursaria* Chlorella virus 1 (PBCV-1)** | **A** | **NP_048554** | **372** | **38** |
| Yellowstone lake phycodnavirus 1 | A | YP_009174737 | 366 | 37 |
| **Yellowstone lake phycodnavirus 2** | **A** | **YP_009174582** | **376** | **38** |
| Yellowstone lake phycodnavirus 3 | A | YP_009174273 | 362 | 36 |
| Dishui Lake phycodnavirus 2 | A | QIG59483 | 398 | 46 |
| Dishui Lake phycodnavirus 3 | A | QIG59731 | 397 | 45 |
| Dishui Lake phycodnavirus 4 | A | QIG59965 | 402 | 41 |
| Organic Lake phycodnavirus 1 (OLPV-1) | A | ADX05944 | 370 | 26 |
| *Cafeteria roenbergensis* virus BV-PW1 (CroV) | I | YP_003969779 | 354 | 33 |
| **Tupanvirus soda lake** | **I** | **QKU35264** | **440** | **100** |
| Tupanvirus deep ocean | I | QKU34009 | 437 | 96 |
| Fadolivirus 1 | I | QKF93844 | 414 | 45 |
| **Klosneuvirus KNV1** | **I** | **ARF12269** | **420** | **43** |
| Terrestrivirus sp. (soil metagenome) | I | AYV75558 | 486 | 48 |
| Yasminevirus sp. GU-2018 | I | VBB18187 | 628 | 48 |
| Murmansk poxvirus | PO | YP_009408354 | 404 | 30 |
| NY_014 poxvirus | PO | YP_009408554 | 402 | 30 |
| Yokapox virus | PO | YP_004821510 | 382 | 29 |

ODC homologues biochemically characterized in the current study are indicated in bold red. The *Paramecium bursaria* chlorella virus-1 (PBCV-1) encodes a homologue of eukaryotic ornithine decarboxylase that has evolved into an arginine decarboxylase. Orders/Families: H, *Herpesviridae*; A, *Algavirales*; I, *Imitervirales*; PO, *Poxviridae*. Protein accession numbers from GenBank are provided. Percentage amino acid identities (% i.d.) are provided for each ODC homologue relative to Tupanvirus soda lake, which is set at 100%.

**Table S2. Chlorovirus-encoded polyamine metabolism genes.** Percentage amino acid identity to *Paramecium bursaria* chlorella virus-1 (PBCV-1).

| Virus | O/ADC | AIH | NCPAH | HSS | SAT |
|---|---|---|---|---|---|
| PBCV-1 | 100 | 100 | 100 | 100 | 100 |
| PBCV KS1B | 98.92 | 96.10 | 97.99 | 99.42 | 85.28 |
| PBCV AN69C | 98.39 | 96.38 | 98.32 | 99.81 | 99.49 |
| PBCV CviKl | 98.12 | 96.38 | 98.32 | 99.61 | 98.98 |
| PBCV CvsA1 | 98.12 | 95.54 | 68.47 | 99.81 | 98.98 |
| PBCV NY2A | 86.29 | 96.66 | 97.31 | 94.31 | 86.80 |
| OSNV5 | 86.56 | n.d. | 89.23 | 96.12 | 84.67 |
| PBCV MA1D | 83.33 | 97.21 | 85.19 | 94.91 | 86.80 |
| PBCV AR158 | 83.33 | 96.94 | 84.51 | 94.70 | 86.29 |
| PBCV CVG-1 | 64.08 | n.d. | 68.14 | 70.48 | 52.58 |
| PBCV Fr5L | 63.00 | n.d. | 66.78 | 69.44 | 52.06 |
| PBCV NW665.2 | 64.08 | n.d. | 66.78 | 70.48 | 51.78 |
| PBCV CVB-1 | 63.81 | deg | 67.80 | 70.27 | 51.83 |
| PBCV OR0704.2.2 | 62.73 | n.d. | 67.46 | 69.44 | 51.55 |
| PBCV CVM-1 | 63.81 | deg | 67.80 | 70.48 | 51.31 |
| PBCV CZ-2 | 62.73 | 63.13 | 67.46 | 69.44 | 52.06 |
| PBCV CVA-1 | 64.08 | deg | 68.71 | 70.69 | 51.31 |
| PBCV AP110A | 63.81 | 62.85 | 68.14 | 70.89 | 51.00 |
| PBCV FR483 | 63.54 | n.d. | 68.47 | 70.48 | 51.55 |
| PBCV Can18-4 | 63.27 | deg | 67.12 | 69.98 | 51.27 |
| PBCV MT325 | 63.00 | 62.85 | 68.14 | 69.98 | 51.78 |
| ATCV Br0604L | 61.28 | 65.36 | 62.67 | 63.28 | 56.54 |
| ATCV TN603.4.2 | 61.00 | 65.64 | 65.07 | 64.03 | 56.02 |
| ATCV GM0701.1 | 60.45 | 66.20 | 64.38 | 64.03 | 54.97 |
| ATCV Canal-1 | 58.77 | 66.20 | 64.26 | 62.79 | 55.50 |
| PBCV NE-JV-1 | 57.99 | n.d. | 66.33 | 69.42 | 50.00 |
| ATCV Can0610SP | 61.00 | 67.04 | 63.57 | 64.24 | 53.93 |
| ATCV-1 | 61.00 | 66.39 | 62.67 | 63.62 | 54.45 |
| ATCV MN0810.1 | 60.17 | 67.88 | 64.38 | 63.83 | 51.31 |
| ATCV NTS-1 | 60.72 | deg | 63.57 | 64.11 | 53.40 |
| ATCV NE-JV-2 | 61.00 | 66.95 | 62.67 | 64.24 | 54.45 |
| PBCV IL-5-2s1 | 83.33 | 97.21 | 84.51 | 95.11 | 86.29 |
| PBCV MA1E | 98.12 | 96.66 | 98.32 | 99.81 | 98.98 |
| PBCV NYs1 | 83.33 | 96.66 | 84.51 | 94.91 | 86.29 |
| PBCV NE-JV-4 | 98.39 | 96.38 | 97.31 | 98.64 | 100 |
| ATCV OR0704.3 | 61.00 | 66.48 | 63.57 | 64.03 | 54.97 |
| ATCV MO0605SPH | 61.00 | 66.67 | 62.80 | 63.62 | 54.45 |
| PBCV IL3A | 98.39 | 96.38 | 98.32 | 98.64 | 99.49 |
| ATCV NE-JV-3 | 61.00 | 66.30 | 62.67 | 63.83 | 54.45 |
| ATCV WI0606 | 61.00 | 66.30 | 62.80 | 63.83 | 54.45 |

O/ADC, alanine racemase L-ODC homologue exhibiting L-arginine-specific decarboxylase activity (PBCV-1, GenBank protein accession no. NP_048554; 372 aa); AIH, agmatine iminohydrolase/deiminase (NP_048994; 359 aa); NCPAH, *N*-carbamoylputrescine amidohydrolase (NP_048426; 298 aa); HSS, homospermidine synthase (NP_048585; 518 aa); SAT, spermidine *N*-acetyltransferase (NP_049010; 197 aa). PBCV, *Paramecium bursaria* chlorella virus; ATCV, *Acanthocytis turfacea* Chlorella virus; OSNV, Only Syngen Nebraska virus 5. Absence of genes (n.d.), presence of degraded genes (deg).

**Table S3. Viruses encoding arginase homologues.**

| Virus | Family | Protein | Size (a.a) | % i.d. |
|---|---|---|---|---|
| **Klosneuvirus KNV1** | **I** | **ARF11926** | **276** | **100** |
| Fadolivirus 1 | I | QKF93541 | 278 | 59 |
| *Chrysochromulina ericina* virus | A | YP_009173394 | 264 | 33 |
| Mimiviridae sp. ChoanoVI | I | QDY52109 | 277 | 32 |
| Indivirus ILV-1 | I | ARF09683 | 368 | 33 |
| Fadolivirus 1 | I | QKF93691 | 396 | 30 |
| Barrevirus sp. | I | AYV77313 | 396 | 22 |
| Catovirus_2_122 | I | ARF09173 | 378 | 19 |
| *Bodo saltans* virus | I | ATZ80880 | 401 | 22 |
| Yasminevirus sp. GU-2018 | I | VBB17826 | 563 | 21 |
| Harvfovirus sp. | I | AYV81278 | 336 | 16 |
| Hyperionvirus8_14 | I | AYV83530 | 345 | 22 |

Arginase homologue biochemically characterized in the current study is indicated in bold red. Orders: A, *Algavirales*; I, *Imitervirales*. Protein accession numbers from GenBank are provided. Percentage amino acid identities relative to Klosneuvirus KNV1 arginase are shown (% i.d.).

**Table S4. Phage and virus genomes encoding biochemically functional spermidine *N*-acetyltransferase (SAT/SpeG) homologues.**

| Phage/**virus** | Protein | Size (a.a) | % i.d. | % i.d. |
|---|---|---|---|---|
| *Erwinia* phage phiEaH2 | YP_007237670 | 154 | 100 | n.s. |
| *Erwinia* phage vB_EamM_Caitlin | YP_009292176 | 155 | 56 | n.s. |
| *Bacillus* phage SPBc2 | NP_046564 | 177 | n.s. | 100 |
| *Streptococcus* phage phi-m46.1 | CAR95371 | 174 | n.s. | 28 |
| *Klebsiella* phage ST13-OXA48phi12.4 | QBQ71974 | 186 | n.s. | 23 |
| *Lactococcus* phage bIL311 | NP_076796 | 168 | 23 | 25 |
| **Pithovirus** LCPAC302 | QBK91546 | 117 | 28 | n.s. |
| *Escherichia* phage vB_EcoM_Goslar | QBO63906 | 177 | 30 | n.s. |

Biochemical function was confirmed by complementation of *E. coli* BL21Δ*speG* with the corresponding ORF and detection of the dibenzoylated *N*-acetylspermidine by LC-MS. The percentage amino acid identity of each protein is presented relative to *Erwinia* phage phiEaH2 (100%) or to Bacillus phage SPBc2 (100%). n.s., no detectable similarity by BLASTP and PSIBLAST.

**Table S5. Virus genomes encoding biochemically functional acetylpolyamine amidohydrolases (APAHs) or homologues.**

| Virus | Protein | Size (a.a) | % i.d. |
|---|---|---|---|
| Klosneuvirus KNV1 | **ARF11327** | 345 | 100 |
| Tupanvirus soda lake | **QKU35181** | 354 | 38 |
| Hyperionvirus sp. | **AYV83810** | 351 | 36 |
| Terrestrivirus sp. | AYV75987 | 376 | 31 |
| Yasminevirus sp. GU-2018 | VBB18253 | 433 | 30 |
| Catovirus CTV1 | ARF08589 | 355 | 37 |
| Edafovirus sp. | AYV78408 | 355 | 38 |
| Fadolivirus 1 | QKF93675 | 350 | 51 |
| Tupanvirus deep ocean | QKU33932 | 354 | 36 |
| Satyrvirus sp. | AYV85400 | 364 | 36 |

Proteins biochemically confirmed to possess APAH activity are shown in bold. Others were not tested. Amino acid percentage identity (% i.d.) is shown relative to Klosneuvirus KNV1 (100%).

**Table S6. Viruses encoding translation factor eIF5a homologues.**

| Virus | Order | Protein | Size (a.a) | % i.d. |
|---|---|---|---|---|
| Tupanvirus deep ocean | I | QKU33581 | 152 | 100 |
| Hyperionvirus | I | AYV83010 | 151 | 39 |
| Harvfovirus | I | AYV81692 | 159 | 31 |
| Terrestrivirus | I | AYV76063 | 145 | 31 |
| Fadolivirus | I | QKF93766 | 150 | 28 |
| *Aureococcus anophagefferens* virus | A | YP_009052187 | 143 | 41 |
| Orpheovirus IHUMI-LCC2 | A | YP_009448366 | 150 | 33 |

I, *Imitervirales*; A, *Algavirales*; Protein accession numbers from GenBank are shown. Amino acid percentage identities are shown relative to Tupanvirus deep ocean (100%).

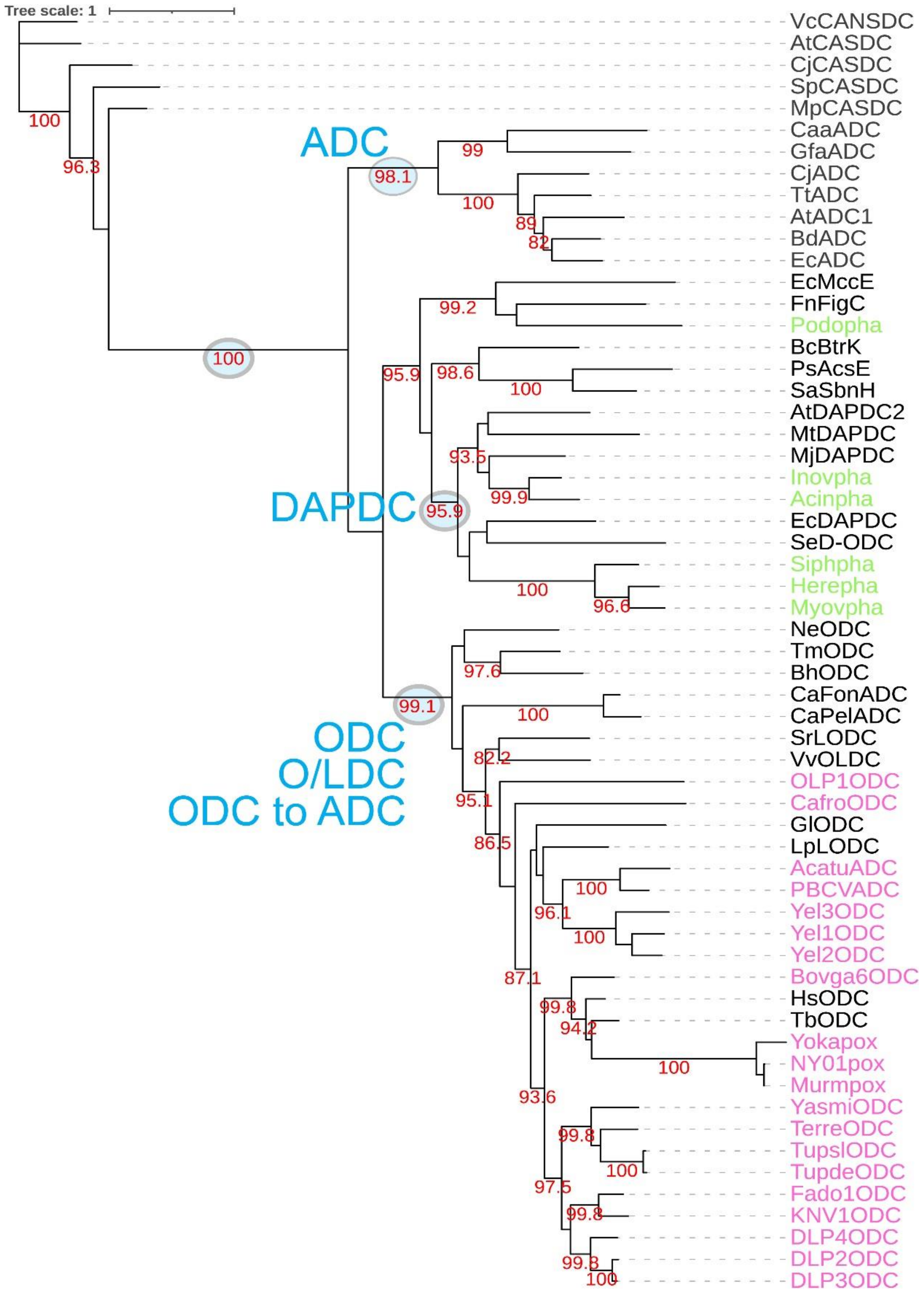


**Fig. S1.** A Maximum Likelihood tree of alanine racemase-fold decarboxylases including virus L-ornithine decarboxylase homologues. The approximately 80 amino acid insertion in the long form ADCs relative to the other decarboxylases was removed, and all N- and C-termini sequences were trimmed to facilitate the alignment. One thousand ultrafast bootstrap values are shown as percentage figures for support above 80%.

**Fig. S1. (cont.)** The main enzymatic functions indicated are: CASDC/CANSDC, carboxyspermidine/carboxynorspermidine decarboxylase; ADC, ancestral short-form and derived long-form arginine decarboxylases; DAPDC, *meso*-diaminopimelate decarboxylase; ODC, L-ornithine decarboxylase; LODC, OLDC, bifunctional L-lysine/L-ornithine decarboxylase. Highly supported clades corresponding to defined enzymatic functions are indicated by circled bootstrap values and the corresponding enzymatic functions. Virus-encoded ODC homologues are shown in pink: CafroODC, *Cafeteria roenbergensis* virus BV-PW1 (YP_003969779); AcatuODC (this is likely to be an ADC evolved from ODC), *Acanthocystis turfacea* Chlorella virus NE-JV-2 (AGE56788); PBCVADC (an ADC evolved from ODC) *Paramecium bursaria* Chlorella virus 1 (NP_048554); Yel3ODC, Yellowstone lake phycodnavirus 3 (YP_009174273); Yel1ODC, Yellowstone lake phycodnavirus 1 (YP_009174737); Yel2ODC, Yellowstone lake phycodnavirus 2 (YP_009174582); Bovga6ODC, Bovine gammaherpesvirus 6 (YP_009041983); YasmiODC, Yasminevirus sp. GU-2018 (VBB18187); TerreODC, Terrestrivirus2_66 (AYV75558); TupsoODC, Tupanvirus soda lake (QKU35264); TupDe, Tupanvirus deep ocean (QKU34009); Fado1ODC, Fadolivirus 1 (QKF93844); KNV1ODC, Klosneuvirus KNV1 (ARF12269); DLP4ODC, Dishui Lake phycodnavirus 4 (QIG59965); DLP2ODC, Dishui Lake phycodnavirus 2 (QIG59483); DLP3ODC, Dishui Lake phycodnavirus 3 (QIG59731); Murmpox, Murmansk poxvirus (YP_009408354), distant homologue of ODC; NY01pox, NY_014 poxvirus (YP_009408554), distant homologue of ODC; Yokapox, Yokapox virus (YP_004821510), distant homologue of ODC. Phage-encoded proteins are shown in green: Acinpha, *Acinetobacter* phage MD-2021a (CAH1009011); Inovpha, Inoviridae sp.(DAO77669); Myovpha, Myoviridae sp. (DAL46253）; Siphpha, Siphoviridae sp. (DAH17773); Herepha, Herelleviridae sp. (DAQ80323); Podopha, Podoviridae sp. (DAI72052); ***Non-viral proteins are***: CaFonADC, *Candidatus Fonsibacter ubiquis*（α-Proteobacteria) ADC evolved from ODC (WP_099339815), this study; CaPelADC, *Candidatus Pelagibacter ubique*（α-Proteobacteria) ADC evolved from ODC (WP_075506504), this study; AtCASDC, *Agrobacterium fabrum* str. C58 (α-Proteobacteria) CASDC (NP_356481); VcCANSDC, *Vibrio cholerae* (γ-Proteobacteria) CANSDC (C6YCI2); CjCASDC, *Campylobacter jejuni* (ε-Proteobacteria) CASDC (WP_002877469); MpCASDC, *Methanolacinia paynteri* (Archaea, Euryarchaeota) CASDC (WP_048150085); GfaADC, *Gramella forsetii* (Bacteroidetes) ancestral short form ADC (YP_863630); CaaADC, *Chloroflexus aurantiacus* (Chloroflexi) ancestral short form ADC (YP_001634722); CjADC, *Campylobacter jejuni* (ε-Proteobacteria) long form ADC (WP_002871443); TtADC, *Thermus thermophilus* (Deinococcus-Thermus) long form ADC (AAS81619); AtADC1, *Arabidopsis thaliana* (Eukaryota, Viridiplantae) long form ADC (AAB09723); EcADC, *Escherichia coli* (γ-Proteobacteria) long form ADC (AAA24646); BdADC, *Bacteroides dorei* (Bacteroidetes) long form ADC (AII67066); SeD-ODC, *Salmonella enterica* subsp. *enterica* serovar Typhimurium (γ-Proteobacteria) D-ODC (AAL21261); EcMccE, *E. coli* (γ-Proteobacteria) microcin C7 protein MccE (YP_006953769); FnFigC, *Francisella tularensis* subsp. *novicida* U112 (γ-Proteobacteria) citrylornithine decarboxylase FigC (ABF50970); BcBtrK, *Bacillus circulans* (Firmicutes) butirosin biosynthesis protein K (Q2L4H3); SaSbnH, *Staphylococcus aureus* (Firmicutes) citryl-L-2,3-diaminopropanoate decarboxylase (AAP82070); PsAcsE, *Pseudomonas syringae* (γ-Proteobacteria) *O*-citryl-L-serine decarboxylase (ELS42881); EcDAPDC, *E. coli* (γ-Proteobacteria) DAPDC (NP_417315); MjDAPDC, *Methanocaldococcus jannaschii* (archaea, euryarchaeota) DAPDC (Q58497); MtDAPDC, *Mycobacterium tuberculosis* (Actinobacteria) DAPDC (2O0T); AtDAPDC2, *Arabidopsis thaliana* (Eukaryota, Viridiplantae) DAPDC (Q94A94); NeODC, *Nitrosomonas europaea* (β-Proteobacteria) L-ODC (WP_011111520);BhODC, *Bartonella henselae*, (α-Proteobacteria) L-ODC (WP_011181090); TmODC, *Thermotoga maritima* (Thermotogae) L-ODC (NP_229669); VvOLDC, *Vibrio vulnificus* (γ-Proteobacteria) L-ornithine/L-lysine decarboxylase (AAO07938); SrLODC, *Selenomonas ruminantium* (Firmicutes) L-lysine/L-ornithine decarboxylase (BAA24923); LpLODC, *Lupinus angustifolius* (Eukaryota, Viridiplantae) L-lysine/L-ornithine decarboxylase (BAK32797); GlODC, *Giardia lamblia* (Eukaryota, Excavata) L-ornithine decarboxylase (EFO63849); TbODC, *Trypanosoma brucei* (Eukaryota, Excavata) L-ODC (AAA30218); HsODC, *Homo sapiens* (Eukaryota, Opisthokonta) L-ODC (AAA59967).

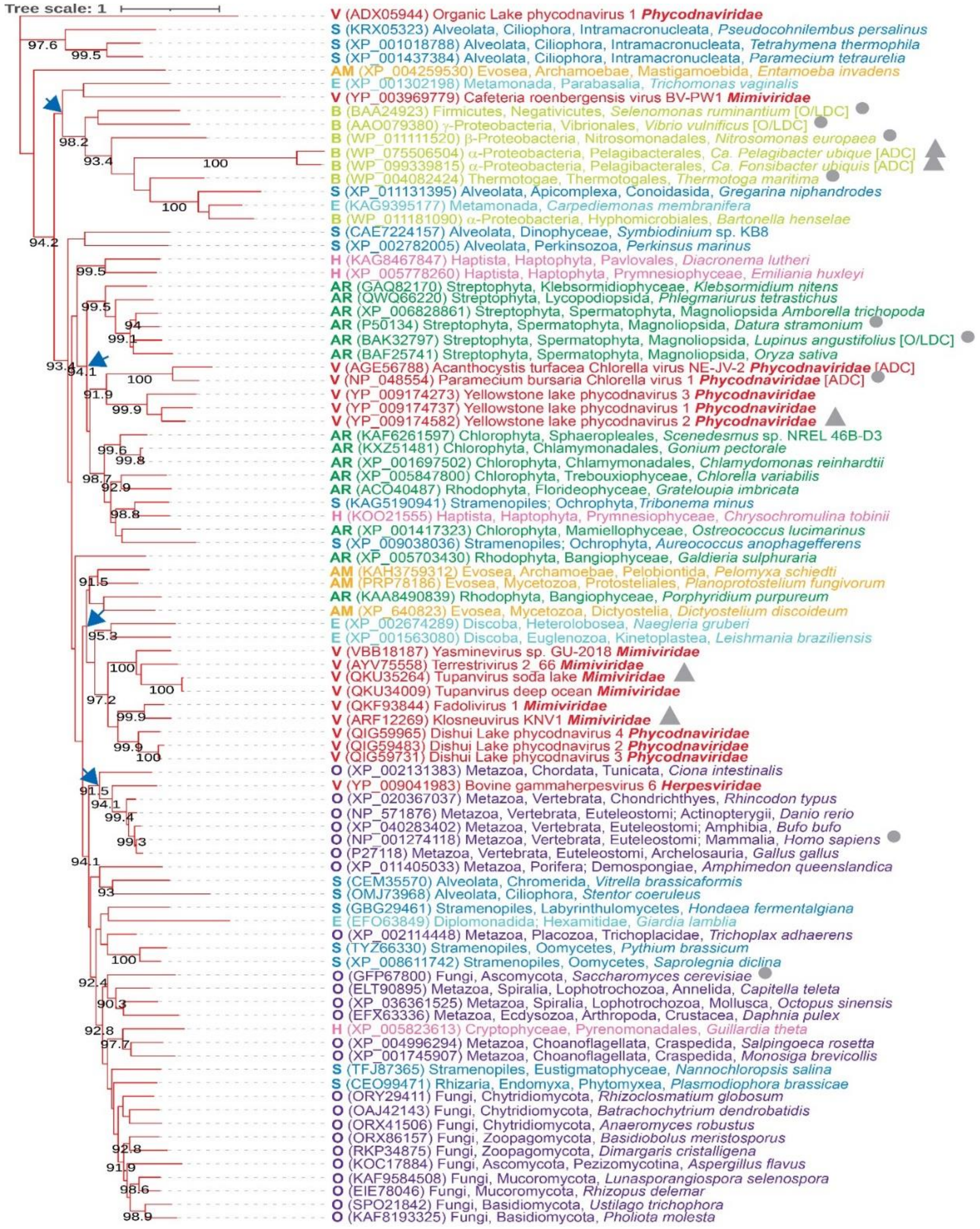


**Fig. S2.** Maximum likelihood phylogenetic tree of ODC homologues from eukaryotes, bacteria and viruses. Branch support greater than 90% is shown (1000 ultrafast bootstraps). Protein GenBank accession numbers are indicated in parentheses. Virus proteins are indicated in red (V), bacteria in light green (B), Excavata in light blue (E), SAR (Stramenopiles, Alveolata, Rhizaria) in dark blue (S), Archaeplastida in dark green (AR), Amoebozoa in orange (AM) and Opisthokonta in purple (O). ODC homologues that have evolved ODC and lysine decarboxylase bifunctionality are indicated by [O/LDC], and ODC homologues that have evolved into arginine decarboxylase are indicated by [ADC]. Distinct clades containing viral proteins are indicated by blue arrows. Previously validated functional activities are indicated by filled grey circles, and activities characterized in the current study, by filled grey triangles.

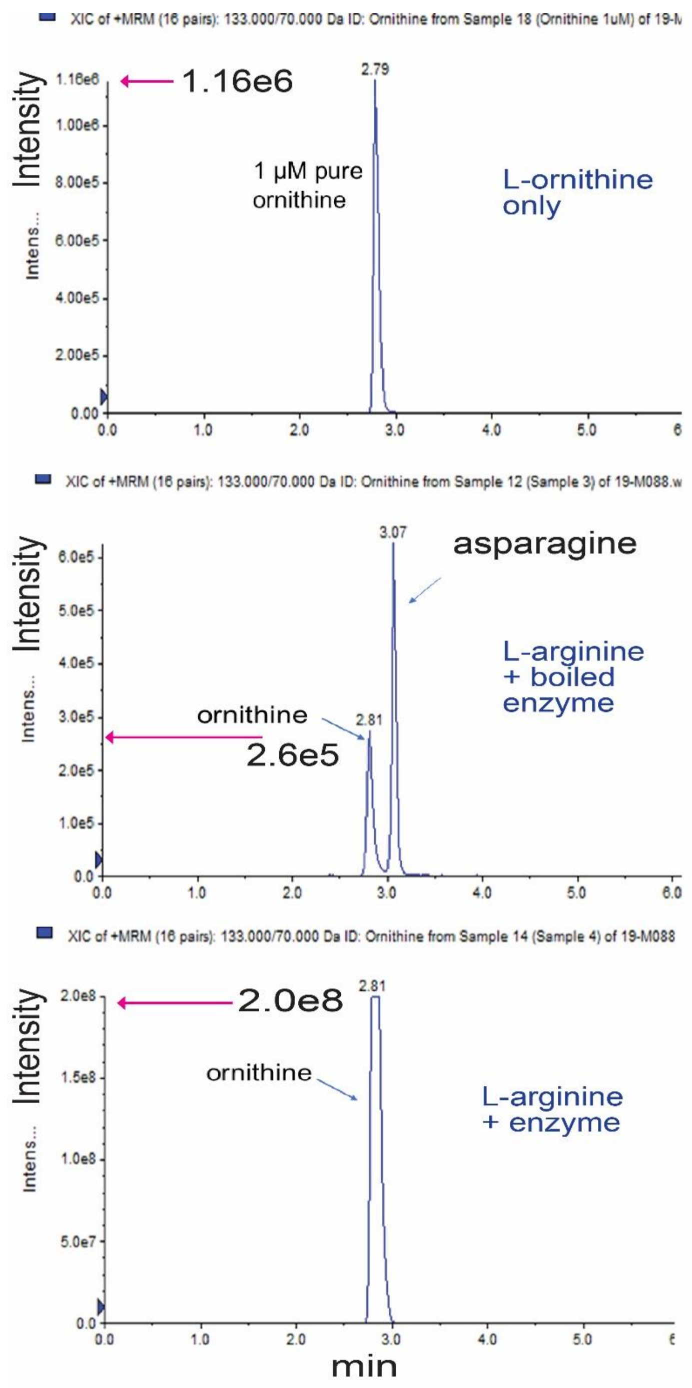


**Fig. S3.** LC-MS/MS analysis of the L-ornithine product (132.16 Da) of Klosneuvirus KNV-1 arginase reaction. Extracted Ion Chromatogram (EIC; 70 Da daughter ion of parental mass 133 Da) of: (upper panel) pure L-ornithine control (1 µM); (middle panel) boiled arginase enzyme with L-arginine (sample loaded represents 100 µM pre-reaction concentration of arginine); (lower panel) arginase enzyme with L-arginine (sample loaded represents 100 µM pre-reaction concentration of arginine). A small amount of contaminating asparagine, which has a similar mass to ornithine (132.12 Da), is present in the L-arginine, seen in the middle panel. Y-axis represents ion intensity, arbitrary units.

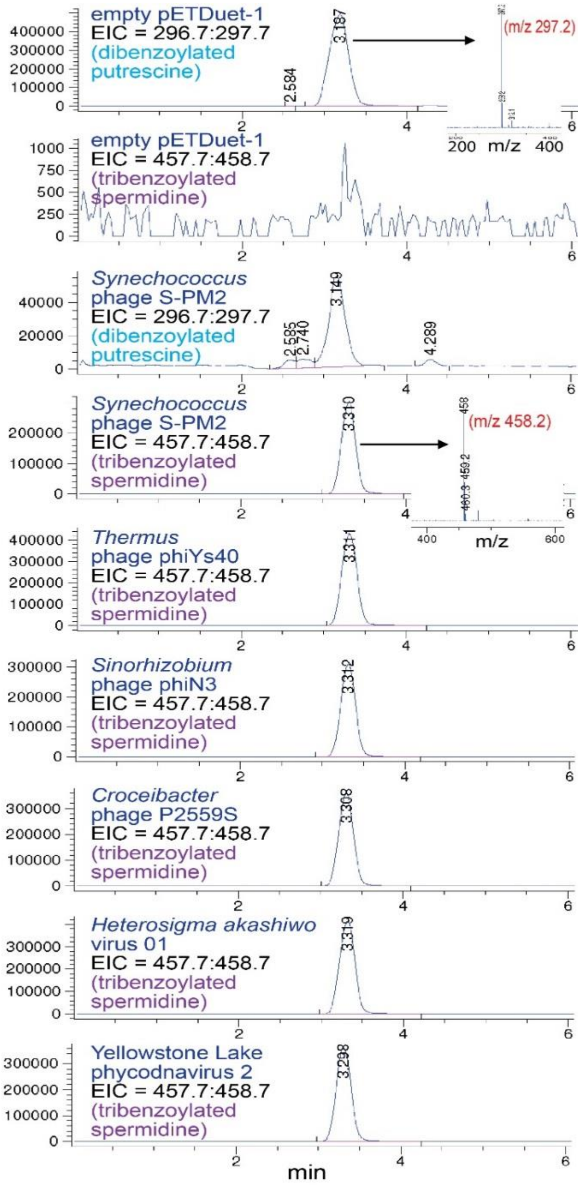


**Fig. S4.** Expression of virus AdoMetDC (SpeD) homologues in an *E. coli* BL21Δ*speD* spermidine-deficient gene deletion strain. LC-MS analysis of benzoylated extracts of *E. coli* BL21*ΔspeD* co-expressing from pETDuet-1 virus and phage AdoMetDC homologue ORFs and the *E. coli* SpdSyn/*speE* ORF. The Extracted Ion Chromatograms corresponding to the mass of tribenzoylated spermidine are shown (EIC = 457.7:458.7). In addition, the EICs corresponding to the mass of dibenzoylated putrescine (EIC = 296.7:297.7) are shown for the BL21Δ*speD* strains expressing the empty pETDuet-1 plasmid and the *Synechococcus* phage S-PM2 AdoMetDC homologue. A representative mass spectrum is shown, for the EIC = 296.7:297.7 peak eluting at 3.187 min in the empty pETDuet-1 strain revealing a mass of m/z 297.2 for putrescine, and for the EIC = 457.7:458.7 peak eluting at 3.3 min in the *Synechococcus* phage S-PM2 AdoMetDC sample, indicating the mass for tribenzoylated spermidine of m/z 458.2. The putrescine peak in the BL21*ΔspeD* strain expressing the *Synechococcus* phage AdoMetDC homologue is reduced by 90% compared to the empty pETDuet-1 putrescine peak, indicating that most of the putrescine is converted to spermidine upon expression of the *Synechococcus* phage AdoMetDC homologue.

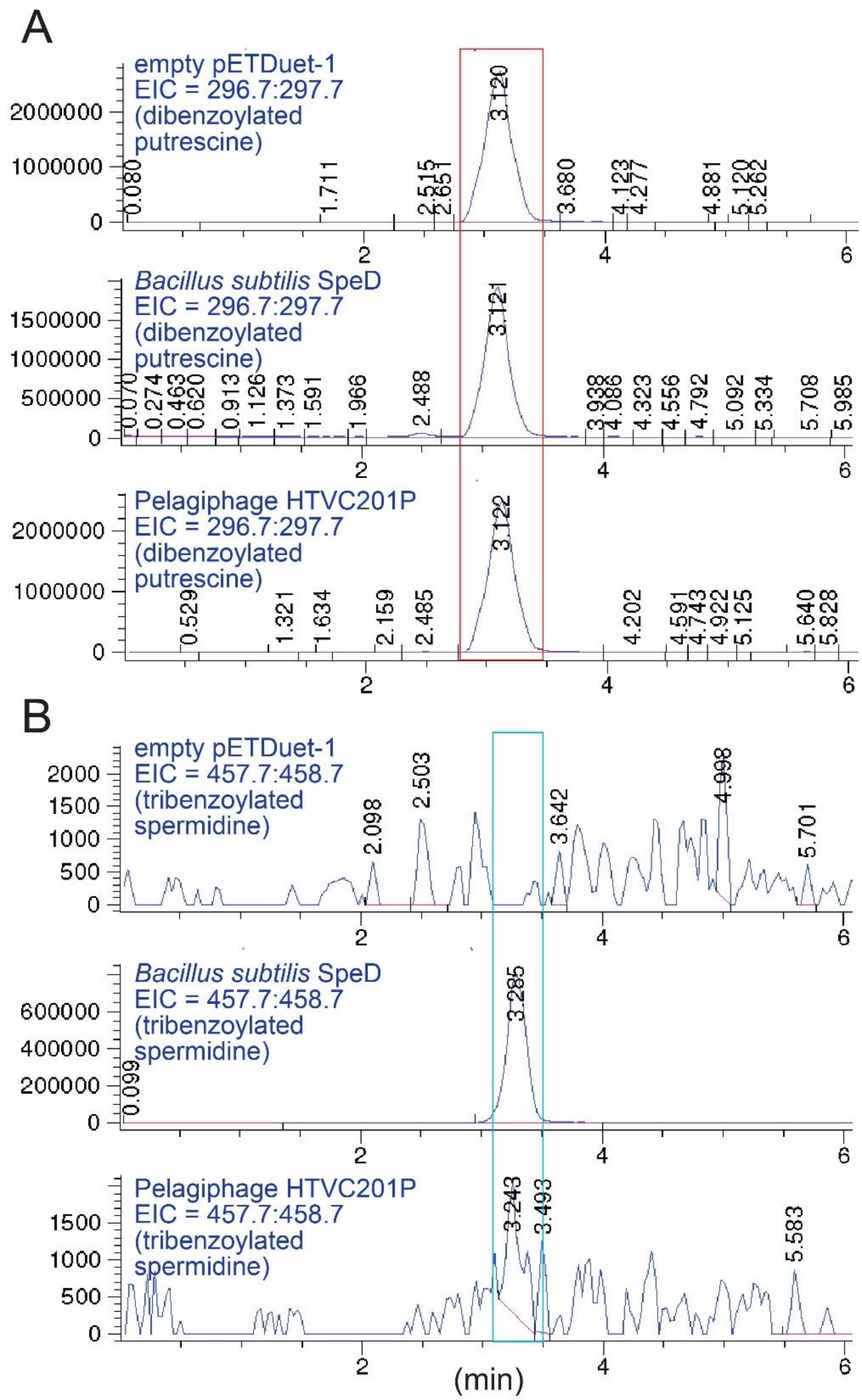


**Fig. S5.** LC-MS analysis of expression of Pelagiphage HTVC201P *S*-adenosylmethionine decarboxylase (*speD*) homologue in spermidine-deficient *E. coli* BL21Δ*speD*. Panels show empty pETDuet-1 plasmid, and pETDuet-1 expressing the *Bacillus subtilis speD* and Pelagiphage HTVC201P *speD* homologue. *A*, Extracted Ion Chromatograms for dibenzoylated putrescine. *B*, Extracted Ion Chromatograms for tribenzoylated spermidine.

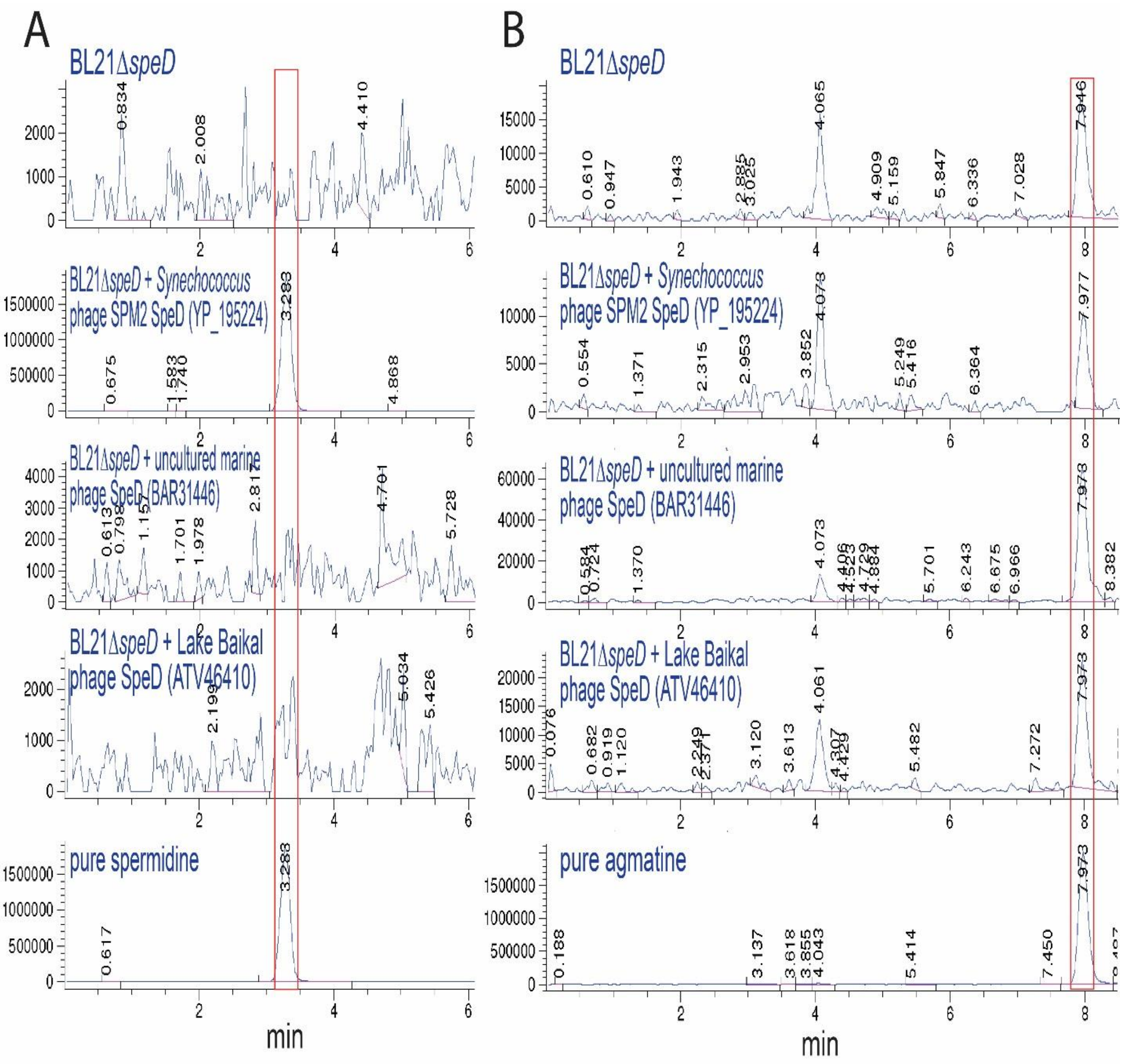


**Fig. S6.** LC-MS analysis of *E. coli* BL21Δ*speD* expressing *S*-adenosylmethionine decarboxylase (SpeD) homologues. Homologues expressed from pETDuet-1 are indicated, followed by the GenBank protein accession number in parentheses. *A*, Extracted Ion Chromatograms for tribenzoylated spermidine (EIC 457.94:458.94) including 100 µM pure spermidine sample. B, Extracted Ion Chromatograms for tribenzoylated agmatine (EIC 442.9;443.9) including 100 µM pure agmatine. The spermidine and agmatine peaks are highlighted in red.

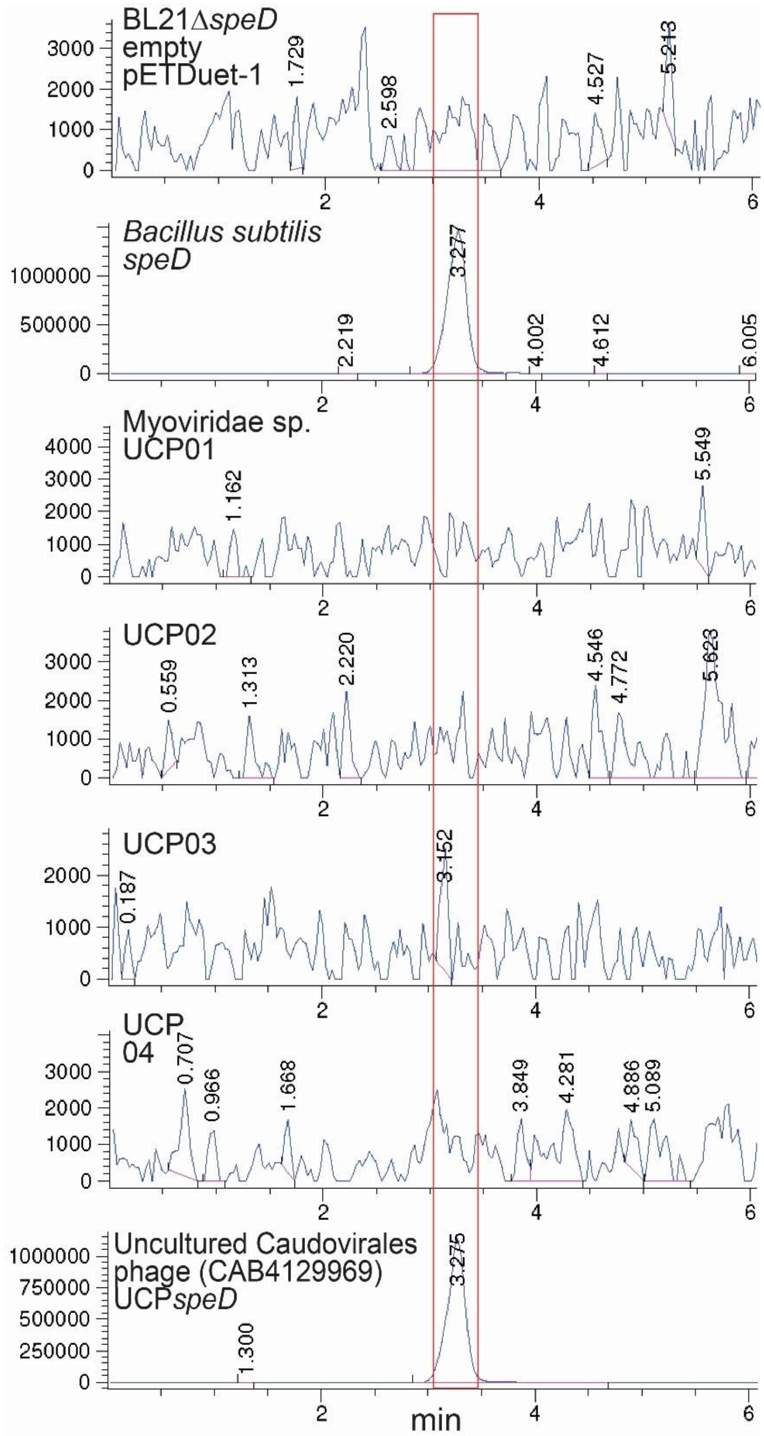


**Fig. S7.** LC-MS analysis of *E. coli* BL21Δ*speD* expressing AdoMetDC (*speD*) homologues from uncultured phages. Spermidine-deficient *E. coli* BL21Δ*speD* was transformed with pETDuet-1 containing the *speD* gene from *Bacillus subtilis*, and *speD* homologues from *Myoviridae* sp. (GenBank protein accession no. QMP83838, UCP01), uncultured Caudovirales phages (CAB5221556, UCP02; CAB4131572; UCP03; CAB41662964; UPC04), and uncultured Caudovirales phage (CAB4129969; UCP*speD*). The extracted ion chromatograms for tribenzoylated spermidine (EIC 457.94:458.94) are shown and the spermidine peak is outlined in red.

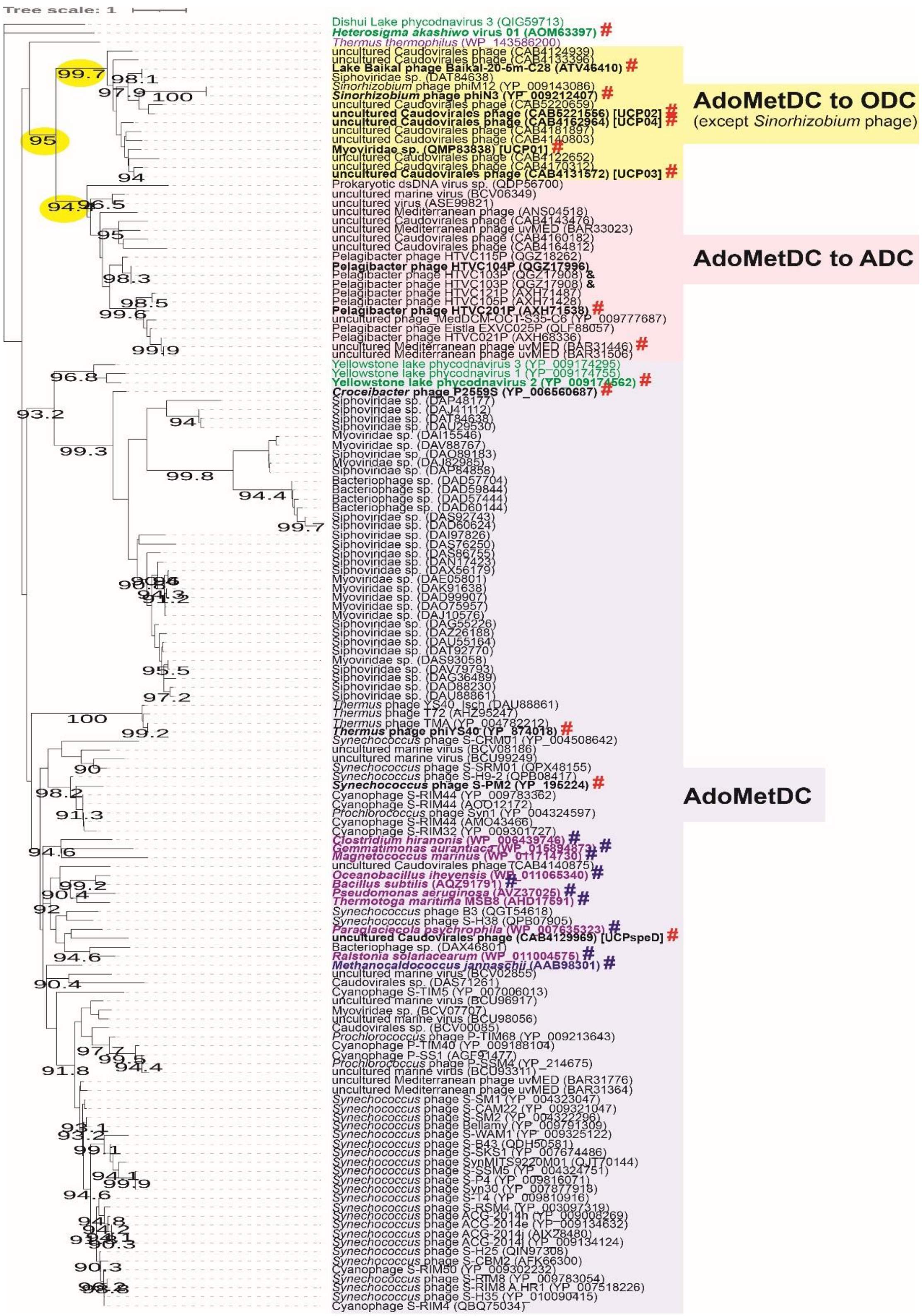


**Fig. S8.** Maximum Likelihood phylogenetic tree of *S*-adenosylmethioine decarboxylase (AdoMetDC/SpeD) homologues.

**Fig. S8. (cont.).** Bacterial AdoMetDC enzymes are shown in purple, archaeal in blue, eukaryotic viruses in green and phage in black text. The GenBank protein accession number is shown in parentheses. Previously published, functionally confirmed bacterial AdoMetDC proteins are shown in bold followed by a blue hash sign, virus and phage AdoMetDC homologues analysed in the current study are shown in bold followed by a red hash sign. Numbers on the ML tree indicate percentage support above 90% based on 1000 ultrafast bootstraps. Inferred functional clades: yellow block, AdoMetDC homologues with ODC activity; pink block, AdoMetDC homologues with ADC activity; mauve block, *bona fide* AdoMetDCs. Two identical proteins from *Synechococcus* phage ACG-2014j represent independent isolates Syn7803US23 (AIX28480) and Syn7803US103 (YP_009134124). Three identical proteins from Cyanophage S-RIM44 represent independent isolates Np_42_0711 (YP_009783362), Np_20_0711 (AOO12172) and W2_07_0710 (AMO43466). The AdoMetDC homologous ORF from Pelagibacter phage HTVC103P (QGZ19908), marked "&", was inadvertently entered twice.

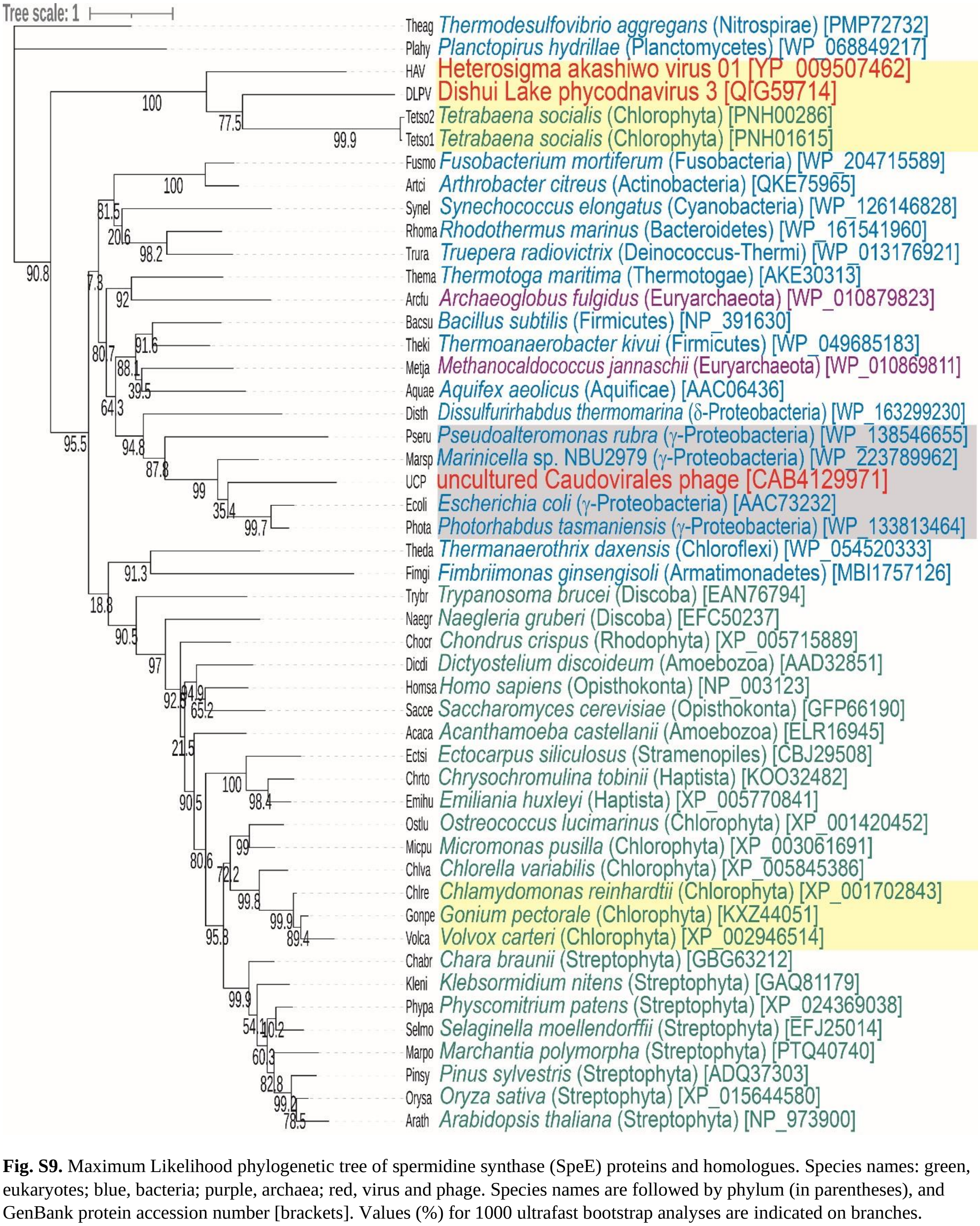


**Fig. S9.** Maximum Likelihood phylogenetic tree of spermidine synthase (SpeE) proteins and homologues. Species names: green, eukaryotes; blue, bacteria; purple, archaea; red, virus and phage. Species names are followed by phylum (in parentheses), and GenBank protein accession number [brackets]. Values (%) for 1000 ultrafast bootstrap analyses are indicated on branches. Volvocine algae are highlighted by yellow blocks, γ-Proteobacteria by a gray block.

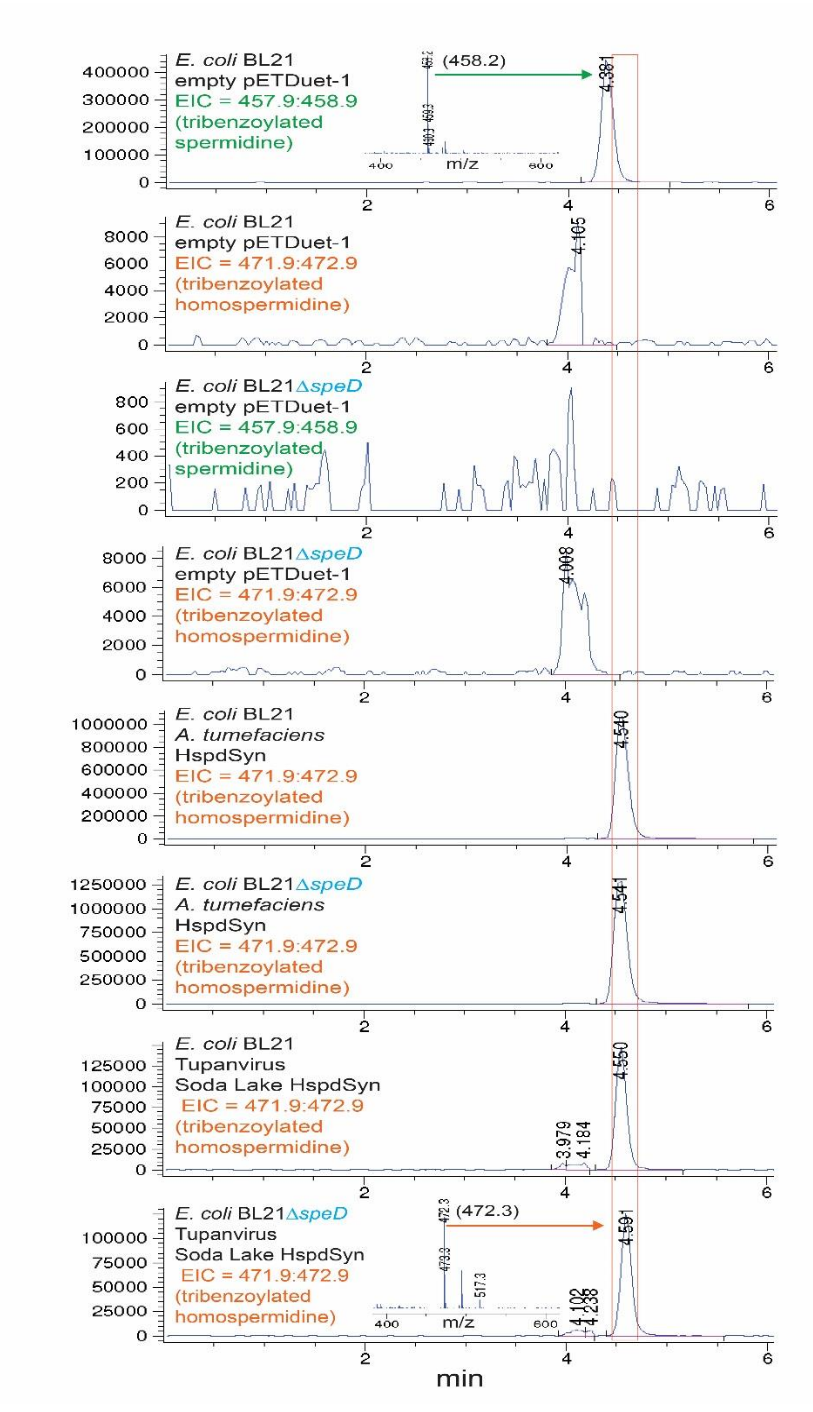


**Fig. S10.** LC-MS analysis of cell extracts from *E. coli* expressing the Tupanvirus soda lake homospermidine synthase homologue. Parental *E. coli* spermidine-replete BL21 or spermidine-deficient BL21Δ*speD* (an *S*-adenosylmethionine decarboxylase gene deletion mutant) expressing empty pETDuet-1 or pETDuet-1 containing the *Agrobacterium tumefaciens* C58 *bona fide* homospermidine synthase (HspdSyn) ORF [AAK89639; 481 aa] or Tupanvirus soda lake HspdSyn homologue ORF [AUL79239; 420 aa]. After induction by IPTG, cell extracts were benzoylated and analyzed by LC-MS. The extracted ion chromatograms (EIC) for tribenzoylated spermidine (457.9:458.9) or tribenzoylated homospermidine (471.9:472.9) are shown. The homospermidine peaks are boxed in red. Mass spectra for spermidine (m/z = 458.2) and homospermidine (m/z = 472.3) are shown for representative peaks.

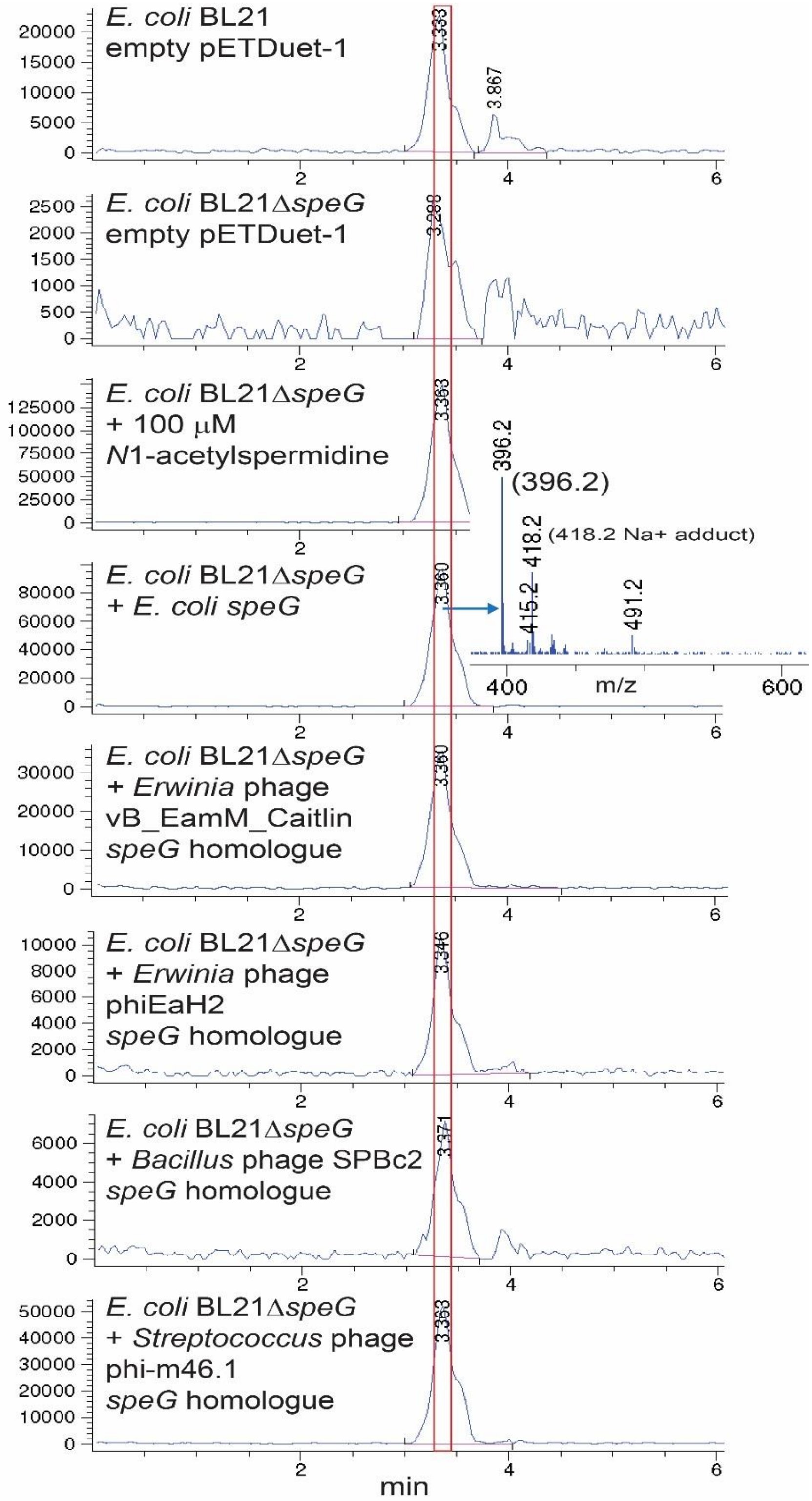


**Fig. S11.** Expression of phage spermidine *N*-acetyltransferase (SAT/SpeG) homologues in an *E. coli* BL21Δ*speG* gene deletion strain. LC-MS analysis of benzoylated extracts of E. *coli* BL21*ΔspeG* gene deletion strain expressing phage SAT homologue ORFs from pETDuet-1. The Extracted Ion Chromatograms (EICs) corresponding to the mass of dibenzoylated N-acetylspermidine are shown (EIC = 396). A representative mass spectrum is shown for the *N*-acetylspermidine peak from *E. coli* BL21*ΔspeG* expressing the *E. coli speG* ORF from pETDuet-1.

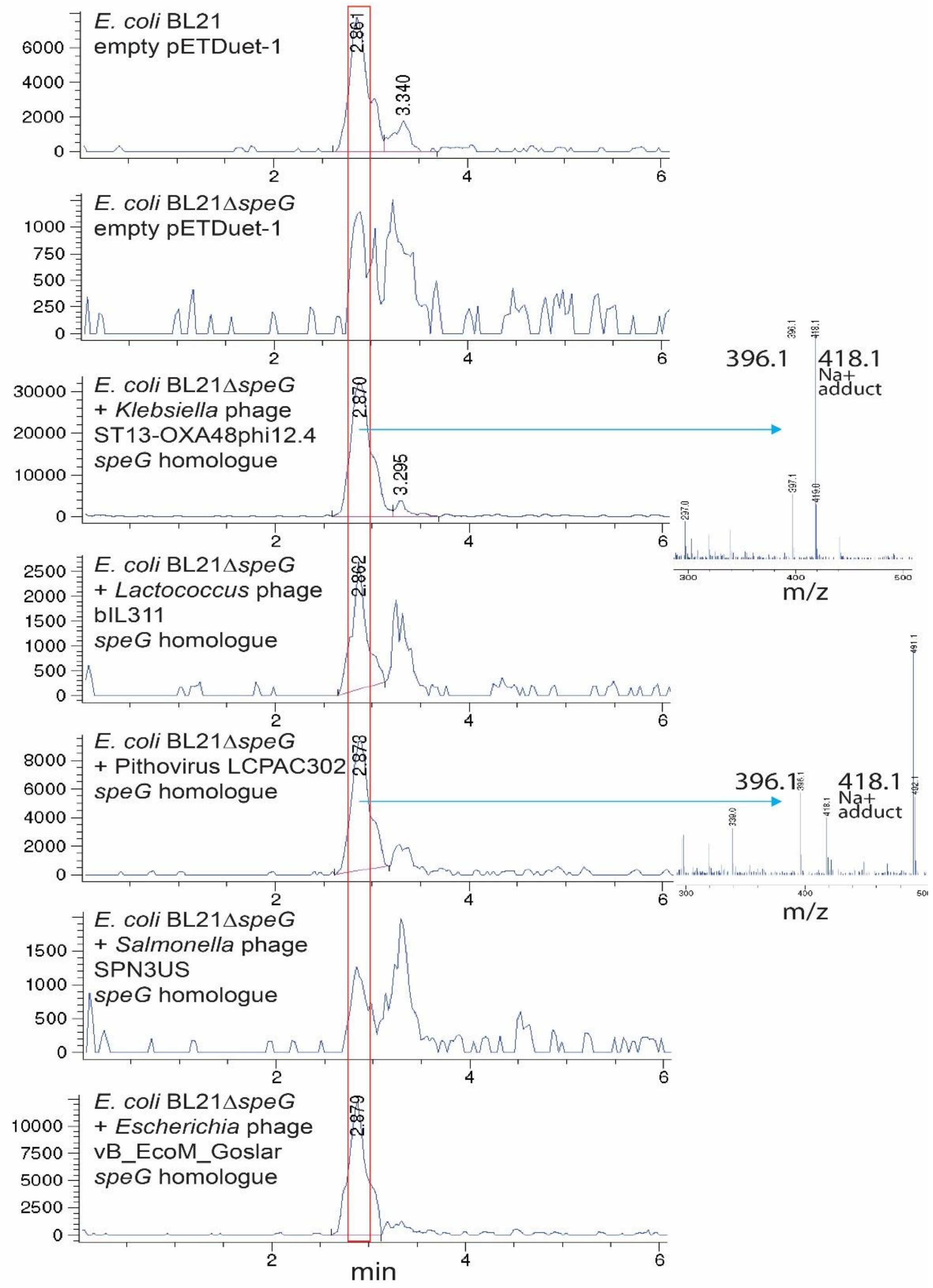


**Fig. S12.** Expression of phage and virus spermidine *N*-acetyltransferase (SAT/SpeG) homologues in an *E. coli* BL21Δ*speG* gene deletion strain. LC-MS analysis of benzoylated extracts of E. *coli* BL21Δ*speG* gene deletion strain expressing phage and virus SAT homologue ORFs from pETDuet-1. The Extracted Ion Chromatograms (EICs) corresponding to the mass of dibenzoylated N-acetylspermidine are shown (EIC = 396). Representative mass spectra are shown for the *N*-acetylspermidine peak from *E. coli* BL21Δ*speG* expressing the *Klebsiella* phage and *Pithovirus speG* homologues from pETDuet-1. No peak for *N*-acetylspermidine above background levels was detected for the *Salmonella* phage SPN3US SAT homologue.

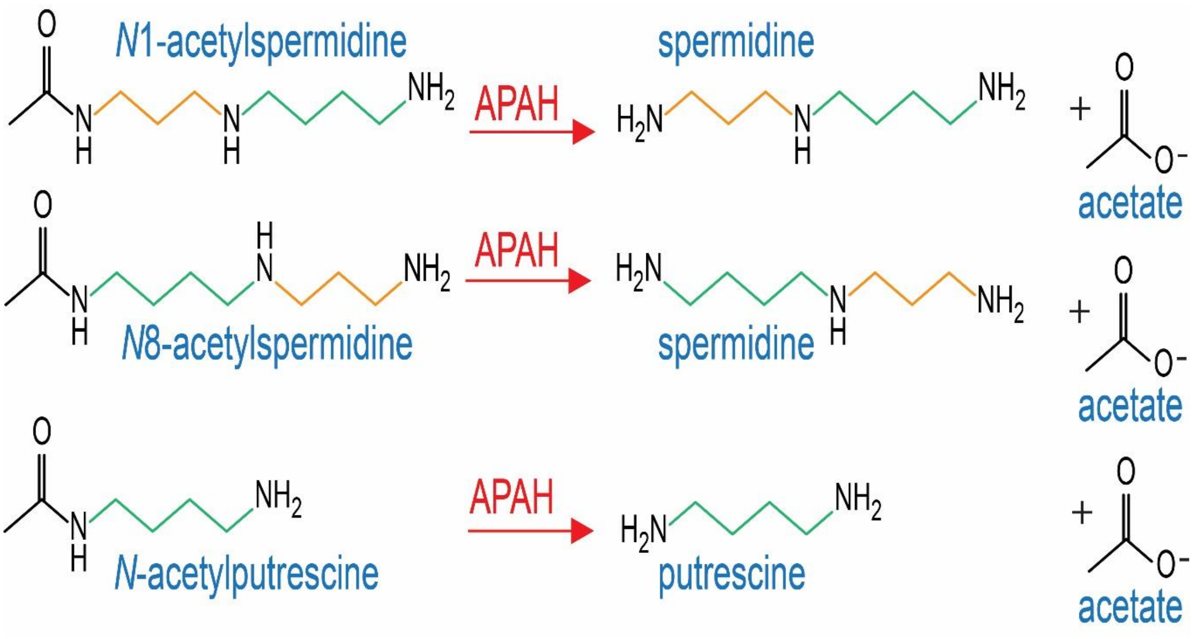


**Fig. S13.** Acetylpolyamine amidohydrolase (APAH) reactions with substrates and products.

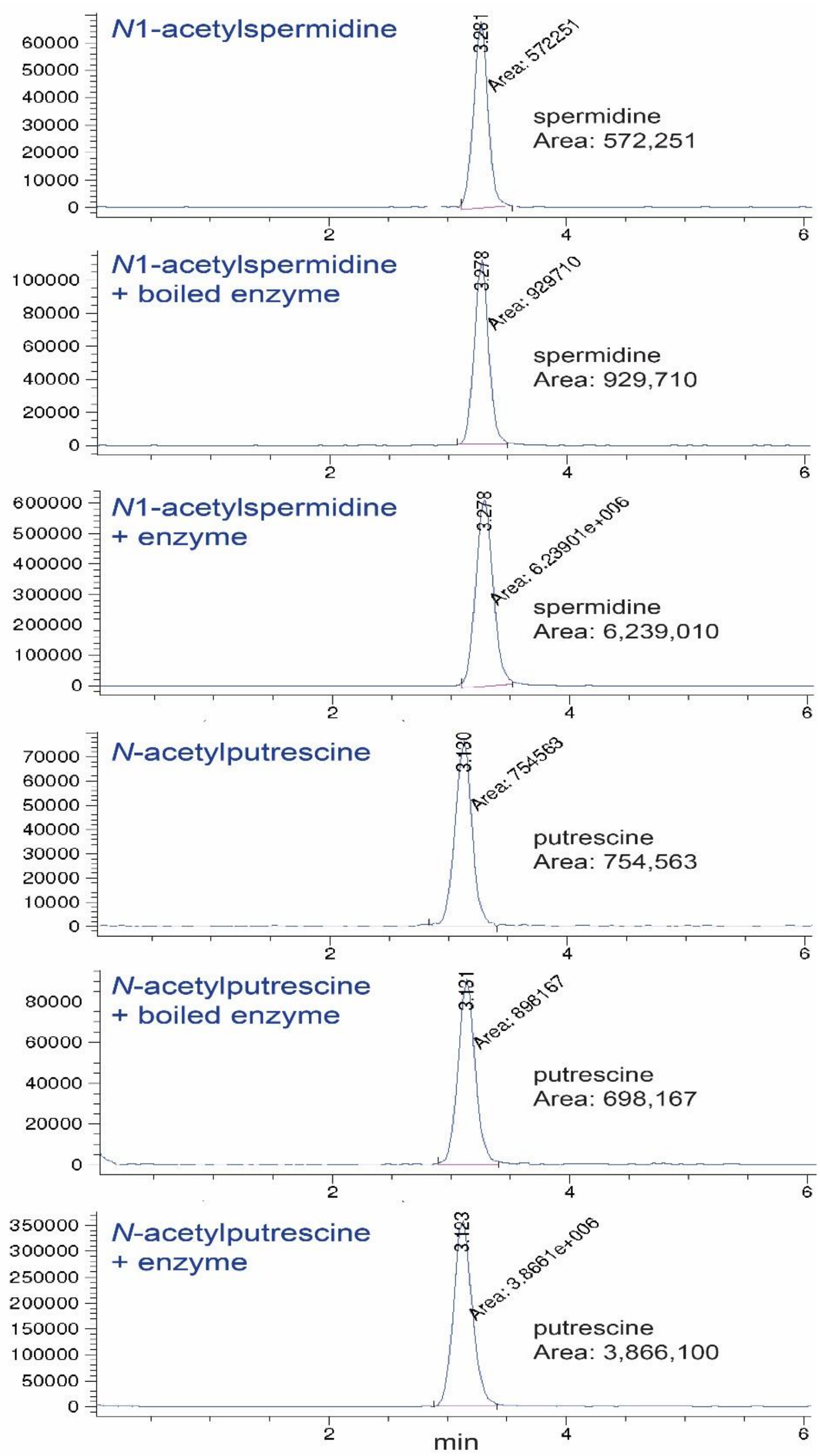


**Fig. S14.** LC-MS analysis of products of Tupanvirus soda lake *N*-acetylpolyamine amidohydrolase (APAH) reactions with $N^1$-acetylspermidine and *N*-acetylputrescine. Extracted Ion Chromatograms (EICs) for tribenzoylated spermidine (EIC = 457.94:498.94) and dibenzoylated putrescine (EIC = 296.85:297.85) are shown. Tupanvirus soda lake APAH protein (1 µM) was assayed with 4 mM $N^1$-acetylspermidine or *N*-acetylputrescine for 60 min at 37 °C and pH 7.4. The reactions without enzyme and with boiled enzyme contain a background level of spermidine or putrescine released from the $N^1$-acetylspermidine and *N*-acetylputrescine. Area, area under the peak. Y axis is an arbitrary scale of relative ion intensity.
